# Supplementary material for: Transient ligand contacts of the intrinsically disordered N-terminus of neuropeptide Y2 receptor regulate arrestin-3 recruitment
Source: Nat Commun. 2025 Sep 19;16:8326. doi: 10.1038/s41467-025-64051-4 (PMC12449476; doi:10.1038/s41467-025-64051-4)
Supplement: Supplementary file 1 — Supplementary Information [file 41467_2025_64051_MOESM1_ESM.pdf]

SUPPLEMENTARY INFORMATION

Transient ligand contacts of the intrinsically disordered N-terminus of neuropeptide Y_2 receptor regulate arrestin-3 recruitment

Anette Kaiser*[1,2], Juan C. Rojas Echeverri[3,4], Asat Baischew[3,4], Maik Pankonin[5], Karl D. Leitner[1], Claudio Iacobucci[3,4,6], Davide Sala[7], Christian Ihling[3,4], Ronny Müller[2], Rok Ferenc[5], Annette G. Beck-Sickinger[2], Peter Schmidt[5], Jens Meiler[7], Peter W. Hildebrand[5], Andrea Sinz*[3,4]

[1] Department of Anesthesiology and Intensive Care, University of Leipzig Medical Center, Liebigstr. 20, 04103 Leipzig, Germany.

[2] Institute of Biochemistry, Faculty of Life Sciences, Leipzig University, Brüderstr. 34, 04103 Leipzig, Germany.

[3] Department of Pharmaceutical Chemistry and Bioanalytics, Institute of Pharmacy, Martin Luther University Halle-Wittenberg, Kurt-Mothes-Str. 3, 06120, Halle (Saale), Germany.

[4] Center for Structural Mass Spectrometry, Martin Luther University Halle-Wittenberg, Kurt-Mothes-Str. 3, 06120, Halle (Saale), Germany.

[5] Institute of Medical Physics and Biophysics, Medical Faculty, Leipzig University, Härtelstr. 16-18, 04107 Leipzig, Germany

[6] Department of Physical and Chemical Sciences, University of L'Aquila, Via Vetoio, L'Aquila, 67100, Italy

[7] Institute for Drug Discovery, Medical Faculty, Leipzig University, Härtelstr. 16-18, 04107 Leipzig, Germany.

*address correspondence to Anette Kaiser (email: anette.kaiser@medizin.uni-leipzig.de; ORCID 0000-0002-5477-201X) and Andrea Sinz (email: andrea.sinz@pharmazie.uni-halle.de; ORCID 0000-0003-1521-4899)

Supplementary Figures

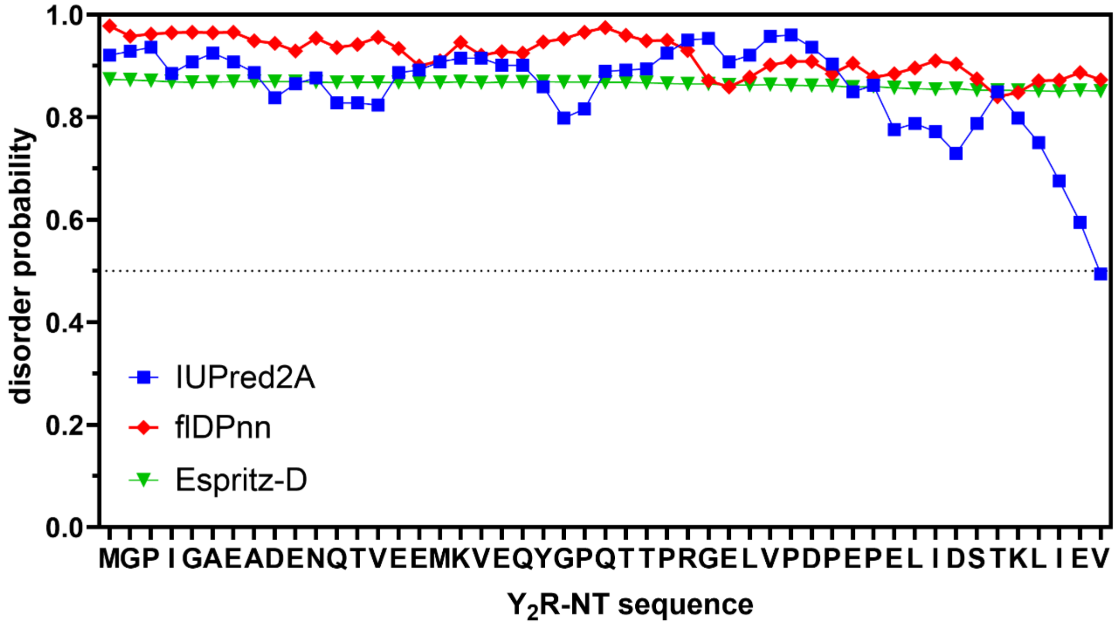

Supplementary Figure 1: Prediction of Y_2R NT disorder by different algorithms. Residues with disorder propensity have a score near 1, while scores <0.5 are likely ordered. Predictions were made using the online servers of IUPred2A (ref[37]) (https://iupred2a.elte.hu/), flDPnn (ref[38]) (http://biomine.cs.vcu.edu/servers/flDPnn/) and Espritz-D (ref[39]) (http://old.protein.bio.unipd.it/espritz/) and consistently show very high disorder probability for Y_2R NT sequence.

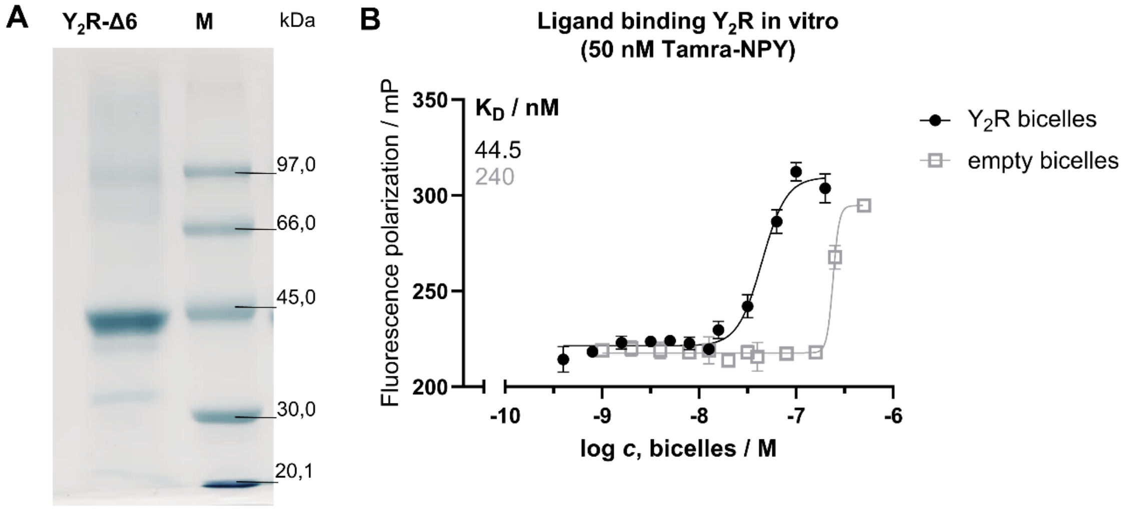

Supplementary Figure 2: Characterization of lipid-reconstituted cysteine-deficient Y_2R (Y_2R-Δ6). A) SDS-PAGE of purified protein. B) Fluorescence polarization measurements at a fixed ligand concentration of 50 nM Tetramethylrhodamine-NPY (Tamra-NPY) confirmes specific ligand binding of Y_2R-loaded bicelles. Data are mean ± SEM of triplicate data. Data are representative examples of two independent experiments.

A

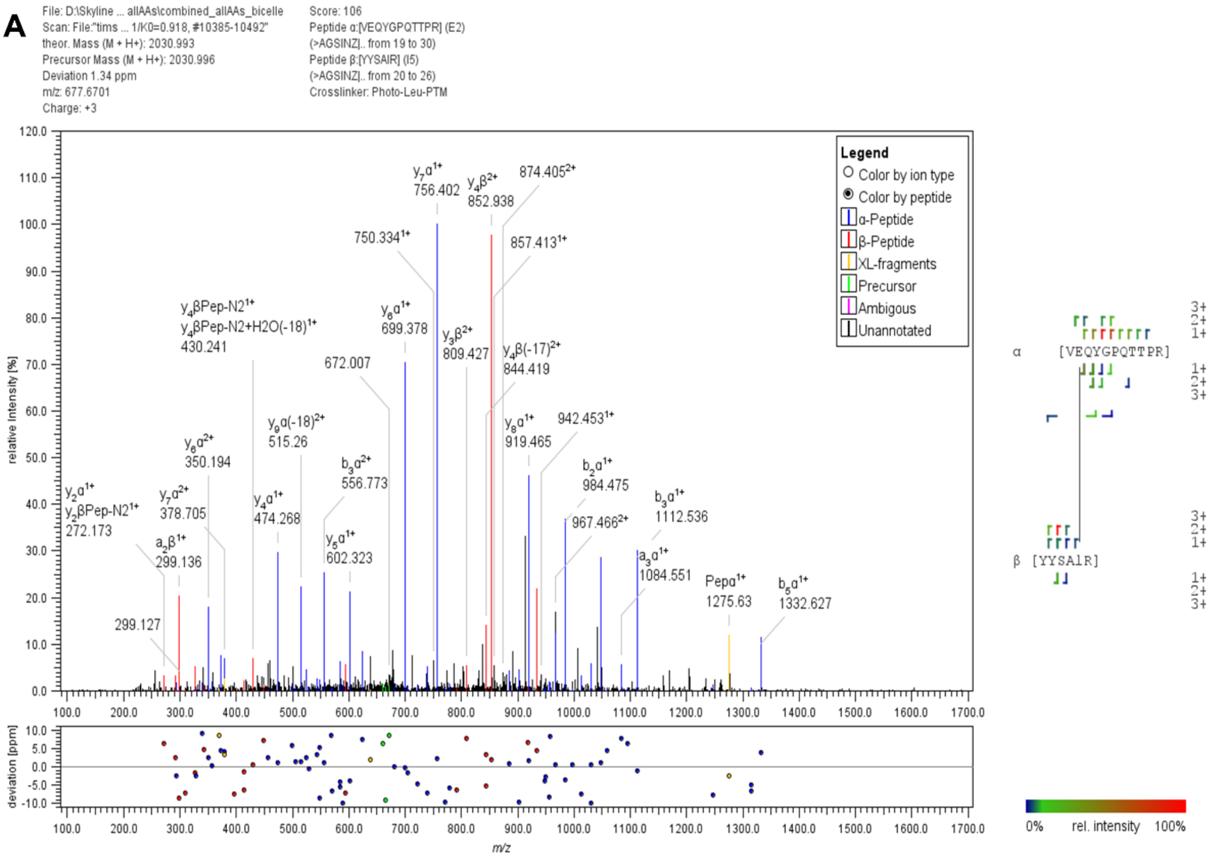
File: D:\Skyline ... allAAs\combined_allAAs_bicelle
Scan: File:"tims ... 1/K0=0.918, #10385-10492"
theor. Mass (M + H+): 2030.993
Precursor Mass (M + H+): 2030.996
Deviation 1.34 ppm
m/z: 677.6701
Charge: +3
Score: 106
Peptide α:[VEQYGPQTTPR] (E2)
(>AGSINZ|.. from 19 to 30)
Peptide β:[YYSAIR] (I5)
(>AGSINZ|.. from 20 to 26)
Crosslinker: Photo-Leu-PTM
Legend
Color by ion type
Color by peptide
α-Peptide
β-Peptide
XL-fragments
Precursor
Ambigous
Unannotated
relative Intensity [%]
deviation [ppm]
m/z
α [VEQYGPQTTPR]
β [YYSAIR]
0%
rel. intensity
100%

B

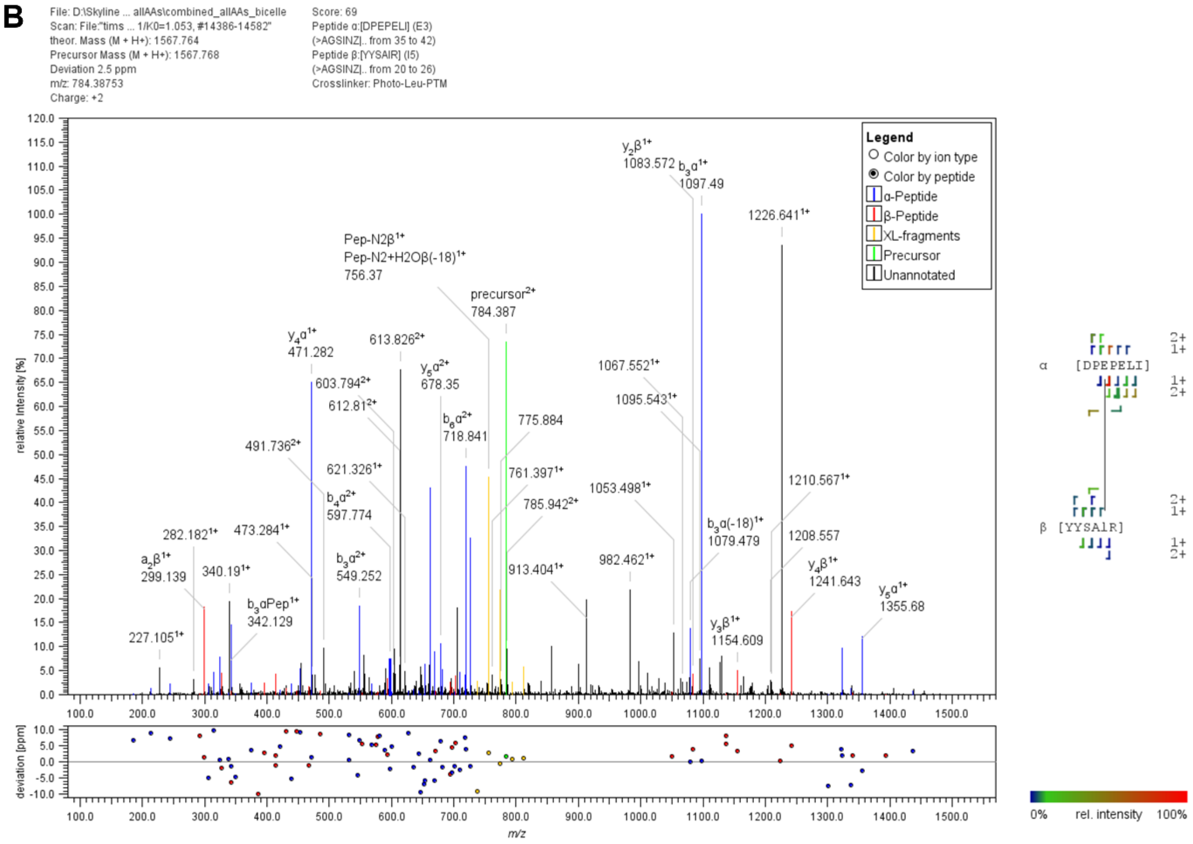
File: D:\Skyline ... allAAs\combined_allAAs_bicelle
Scan: File:"tims ... 1/K0=1.053, #14386-14582"
theor. Mass (M + H+): 1567.764
Precursor Mass (M + H+): 1567.768
Deviation 2.5 ppm
m/z: 784.38753
Charge: +2
Score: 69
Peptide α:[DPEPELI] (E3)
(>AGSINZ|.. from 35 to 42)
Peptide β:[YYSAIR] (I5)
(>AGSINZ|.. from 20 to 26)
Crosslinker: Photo-Leu-PTM
Legend
Color by ion type
Color by peptide
α-Peptide
β-Peptide
XL-fragments
Precursor
Unannotated
relative Intensity [%]
deviation [ppm]
m/z
α [DPEPELI]
β [YYSAIR]
0%
rel. intensity
100%

C

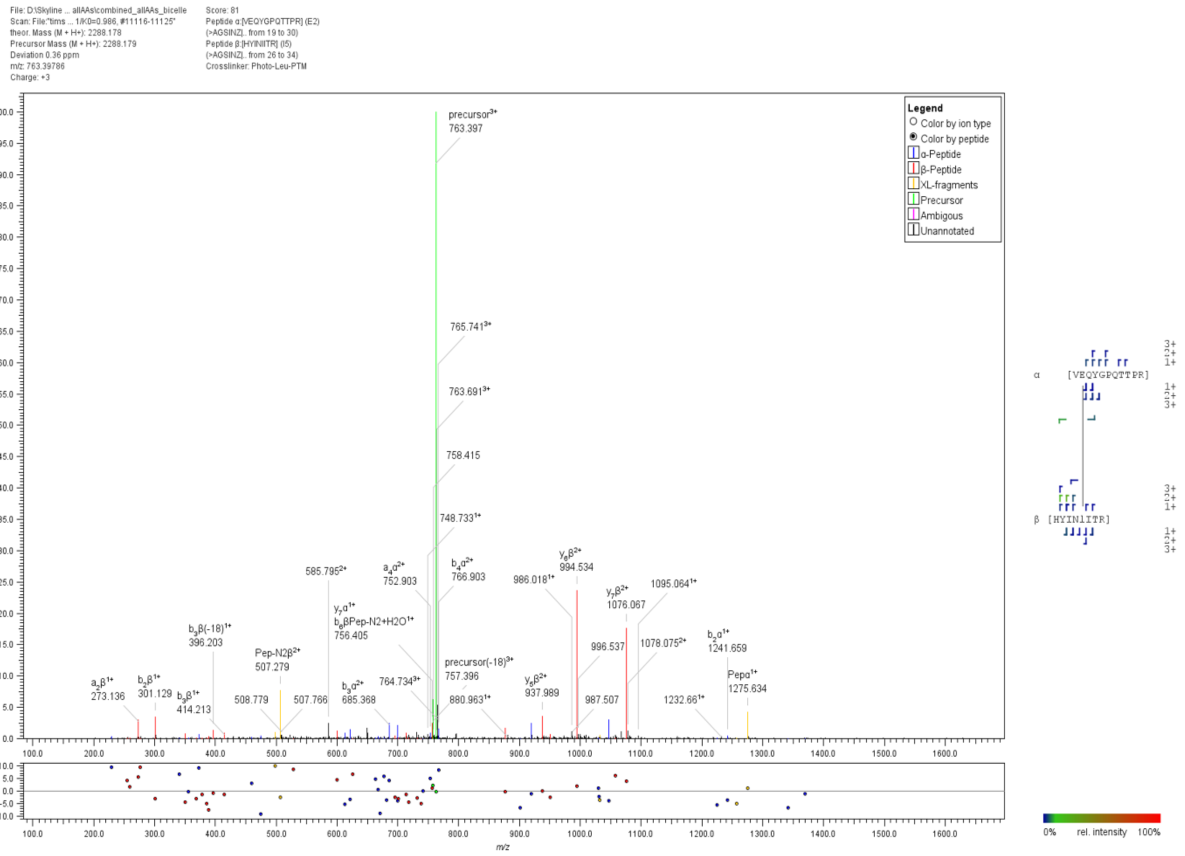

File: D:\Skyline ... allAAs\combined_allAAs_bicelle
Scan: File:"tims ... 1/K0=0.986, #11116-11125"
theor. Mass (M + H+): 2288.178
Precursor Mass (M + H+): 2288.179
Deviation 0.36 ppm
m/z: 763.39786
Charge: +3
Score: 81
Peptide α:[VEQYGPQTTPR] (E2)
(>AGSINZ|.. from 19 to 30)
Peptide β:[HYINIITR] (I5)
(>AGSINZ|.. from 26 to 34)
Crosslinker: Photo-Leu-PTM
Legend
Color by ion type
Color by peptide
α-Peptide
β-Peptide
XL-fragments
Precursor
Ambigous
Unannotated
relative intensity [%]
deviation [ppm]
m/z
0% rel. intensity 100%

D

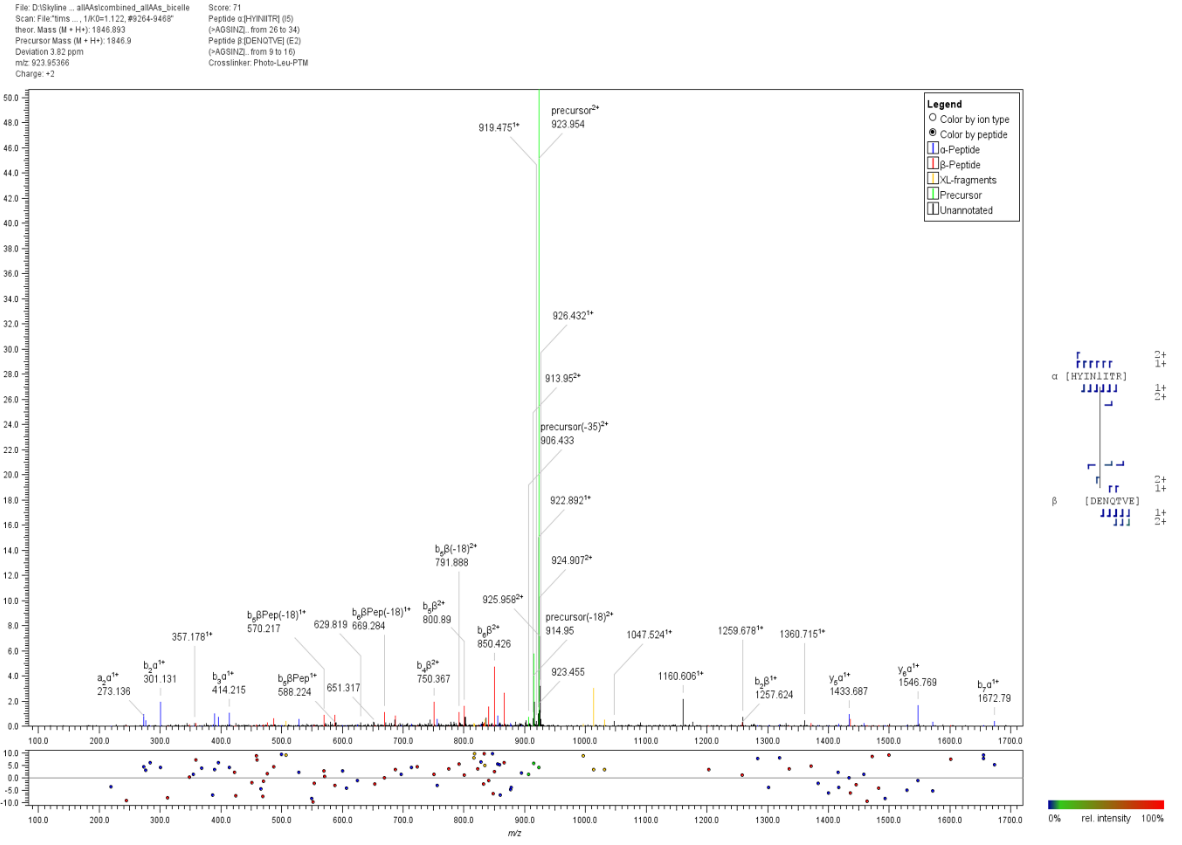

File: D:\Skyline ... allAAs\combined_allAAs_bicelle
Scan: File:"tims ... , 1/K0=1.122, #9264-9468"
theor. Mass (M + H+): 1846.893
Precursor Mass (M + H+): 1846.9
Deviation 3.82 ppm
m/z: 923.95366
Charge: +2
Score: 71
Peptide α:[HYINIITR] (I5)
(>AGSINZ|.. from 26 to 34)
Peptide β:[DENQTVE] (E2)
(>AGSINZ|.. from 9 to 16)
Crosslinker: Photo-Leu-PTM
Legend
Color by ion type
Color by peptide
α-Peptide
β-Peptide
XL-fragments
Precursor
Unannotated
relative intensity [%]
deviation [ppm]
m/z
0% rel. intensity 100%

E

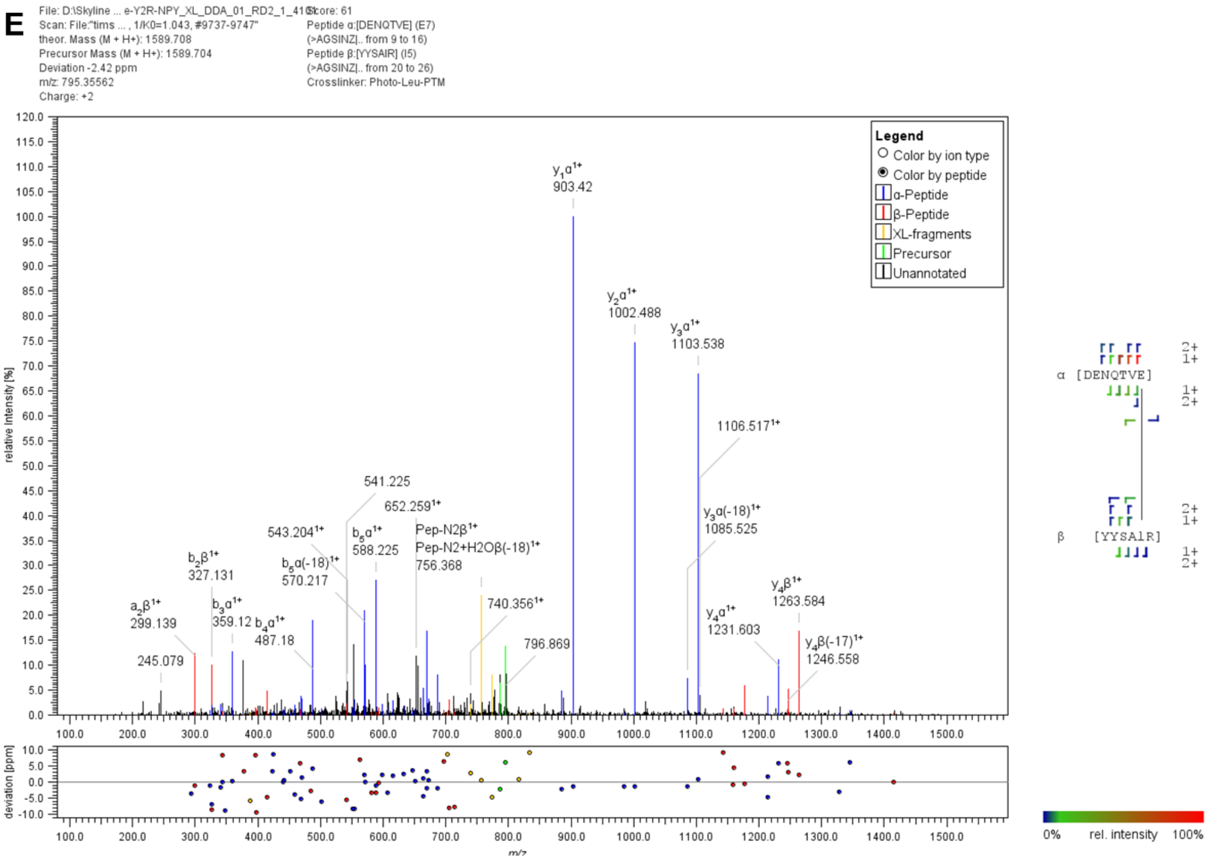
File: D:\Skyline ... e-Y2R-NPY_XL_DDA_01_RD2_1_4103
Scan: File:"tims ... , 1/K0=1.043, #9737-9747"
theor. Mass (M + H+): 1589.708
Precursor Mass (M + H+): 1589.704
Deviation -2.42 ppm
m/z: 795.35562
Charge: +2
Score: 61
Peptide α:[DENQTVE] (E7)
(>AGSINZ|.. from 9 to 16)
Peptide β:[YYSAIR] (I5)
(>AGSINZ|.. from 20 to 26)
Crosslinker: Photo-Leu-PTM
Legend
Color by ion type
Color by peptide
α-Peptide
β-Peptide
XL-fragments
Precursor
Unannotated
relative intensity [%]
deviation [ppm]
m/z
α [DENQTVE]
β [YYSAIR]
0% rel. intensity 100%

F

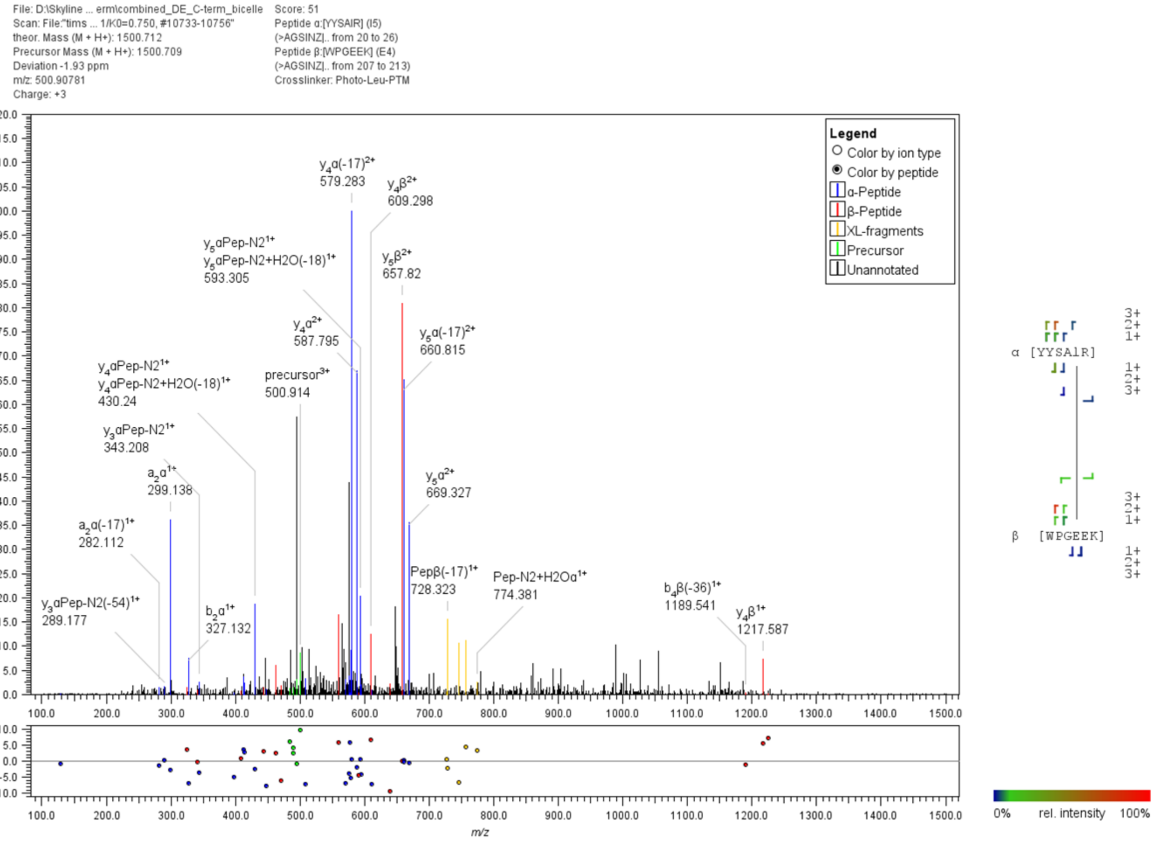
File: D:\Skyline ... erm\combined_DE_C-term_bicelle
Scan: File:"tims ... 1/K0=0.750, #10733-10756"
theor. Mass (M + H+): 1500.712
Precursor Mass (M + H+): 1500.709
Deviation -1.93 ppm
m/z: 500.90781
Charge: +3
Score: 51
Peptide α:[YYSAIR] (I5)
(>AGSINZ|.. from 20 to 26)
Peptide β:[WPGEEK] (E4)
(>AGSINZ|.. from 207 to 213)
Crosslinker: Photo-Leu-PTM
Legend
Color by ion type
Color by peptide
α-Peptide
β-Peptide
XL-fragments
Precursor
Unannotated
relative intensity [%]
deviation [ppm]
m/z
α [YYSAIR]
β [WPGEEK]
0% rel. intensity 100%

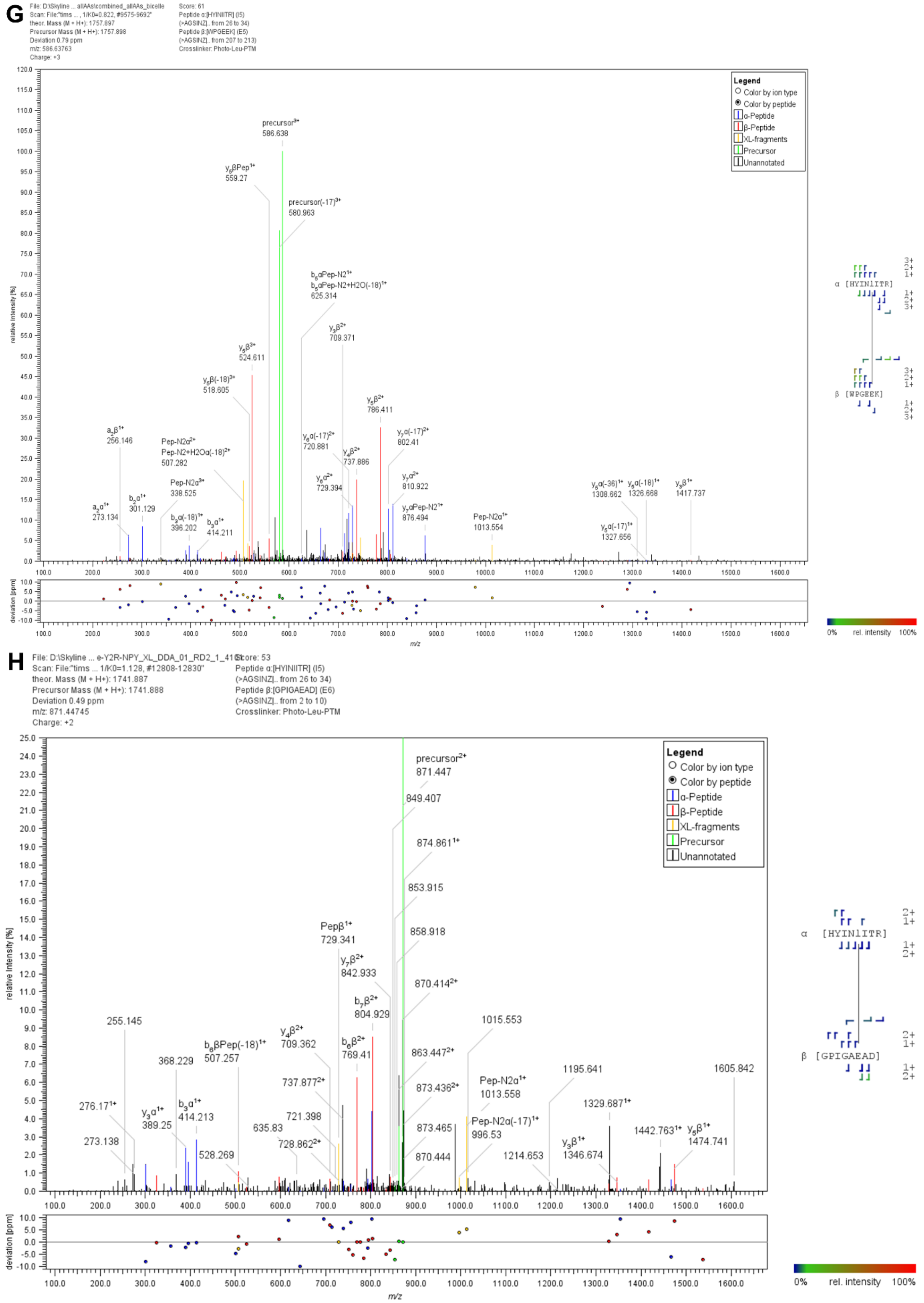

Supplementary Figure 3: Selected fragment ion mass spectra of Y$_2$R-NPY cross-links. Precursor ions are shown in green, b- and y-type ions of cross-linked peptides are presented in blue and red, cleavage of the cross-linker is shown in yellow. A) VEQYGPQTTPR (Y$_2$R aa19–30)-YYSAIR (NPY aa 20–26), B) DPEPELID (Y$_2$R aa35–43)-YYSAIR (NPY aa20–26), C) VEQYGPQTTPR (Y$_2$R aa19–30)-YYSAIR (NPY aa20–26), D) DENQTVE (Y2R aa9–16)-

HYINlITR (NPY aa26–34), E) DENQTVE (Y_2R aa9–16)-YYSAlR (NPY aa20–26), F) WPGEEK (Y_2R aa207–213)-YYSAIR (NPY aa20–26), G) WPGEEK (Y_2R aa207–213)-HYINlITR (NPY aa26–34), H) GPIGAEAD (Y_2R aa2–10)-HYINlITR (NPY aa26–34). Full data is deposited under ProteomeXchange ID PDX051865 (https://proteomecentral.proteomexchange.org/cgi/GetDataset?ID=PXD051865).

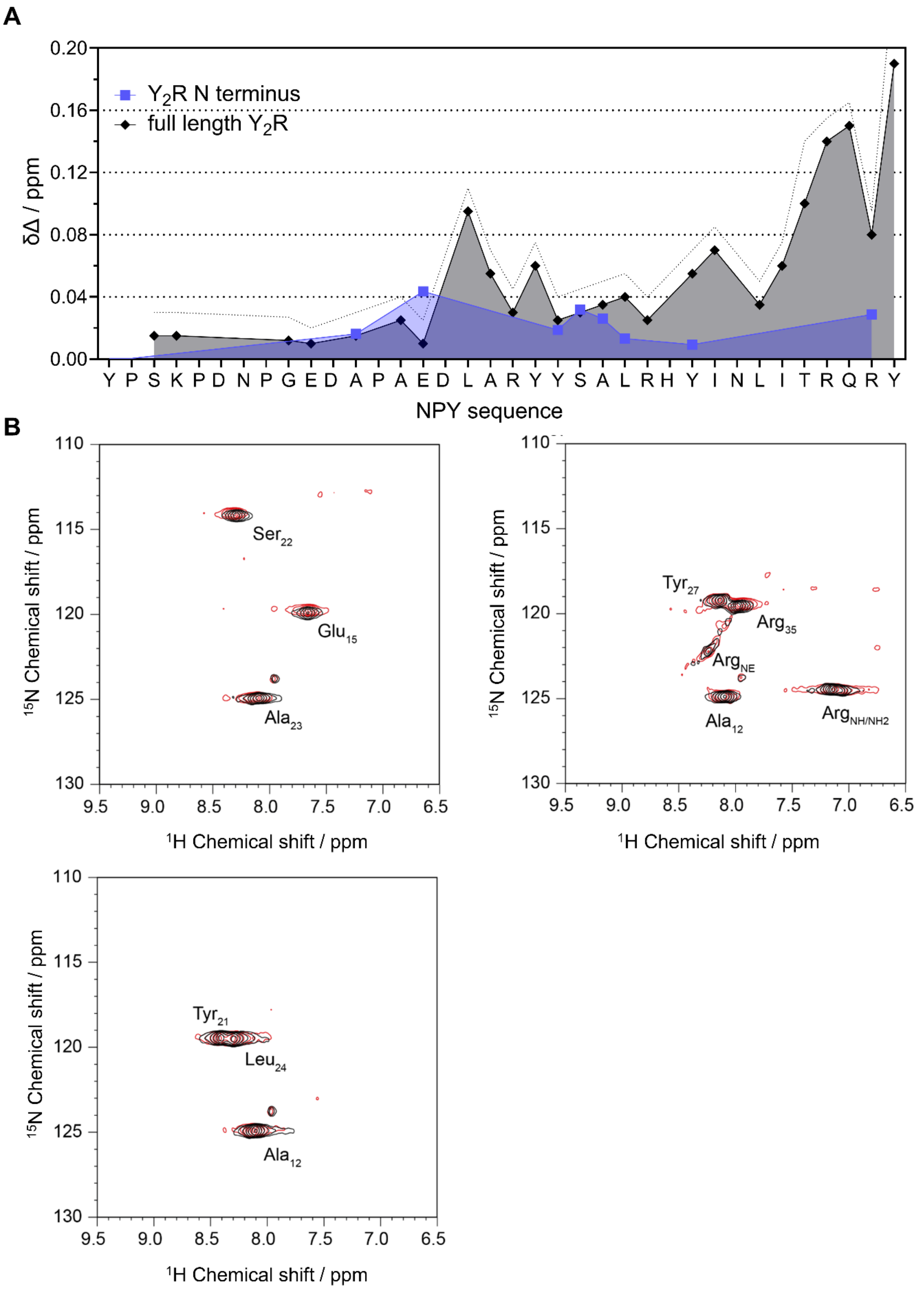

Supplementary Figure 4: Solution-state NMR shows contacts between NPY and Y_2R NT. A) Weighted chemical-shift changes (δΔ) of NPY sites upon addition of the Y_2R NT are shown along the sequence of site-specifically ^{13}C/^{15}N-labeled NPY variants when bound to an (unlabeled) synthetic peptide corresponding to the Y_2R NT (purple). E15^{NPY}, S22^{NPY} and R35^{NPY} show biggest changes in chemical shifts indicating sites of interaction. The interaction pattern of NPY with the N-terminal peptide of Y_2R is different from the full-length Y_2R-bound

conformation (grey, confidence interval as dotted line), taken from ref[28]. B) $^{1}H/^{15}N$ correlation spectra of the three isotopically labeled NPY variants used for interaction studies with synthetic Y_2R NT peptide (black: peptide alone, red: bound to Y_2R NT).

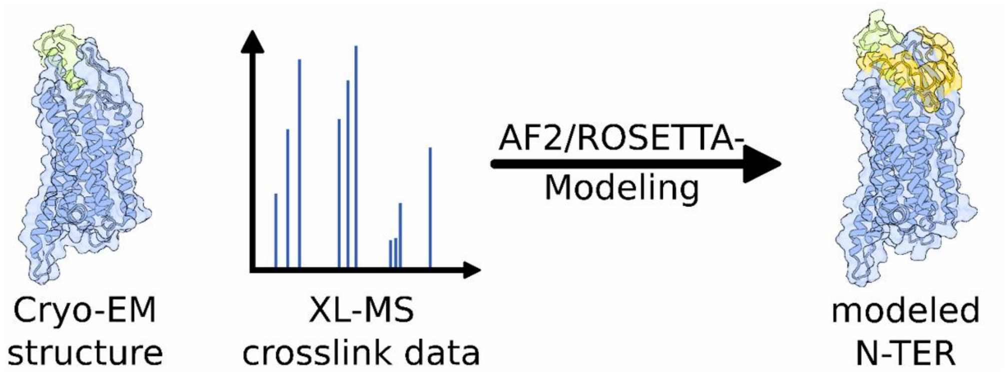

Supplementary Figure 5: Simplified procedure of model generation.

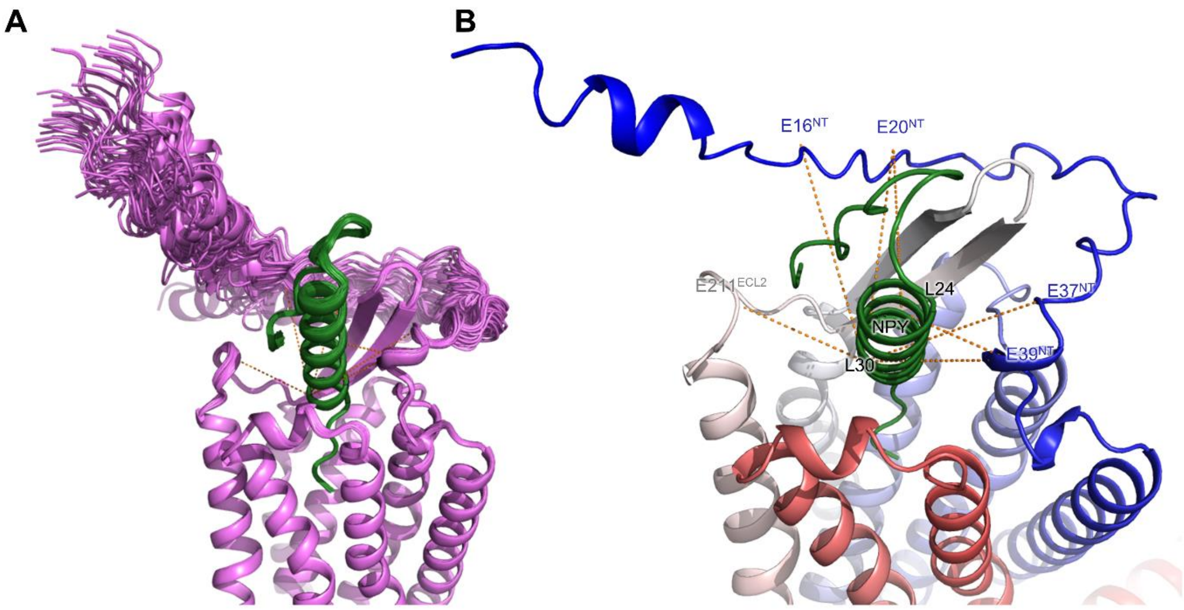

Supplementary Figure 6: Model of full-length Y_2R bound to NPY generated by combining AF2 and Rosetta. A) Overlay of the top 100 ensemble showing the Y_2R in violet, NPY in green. B) NPY/Y_2R contacts derived from XL-MS data mapped onto a single model.

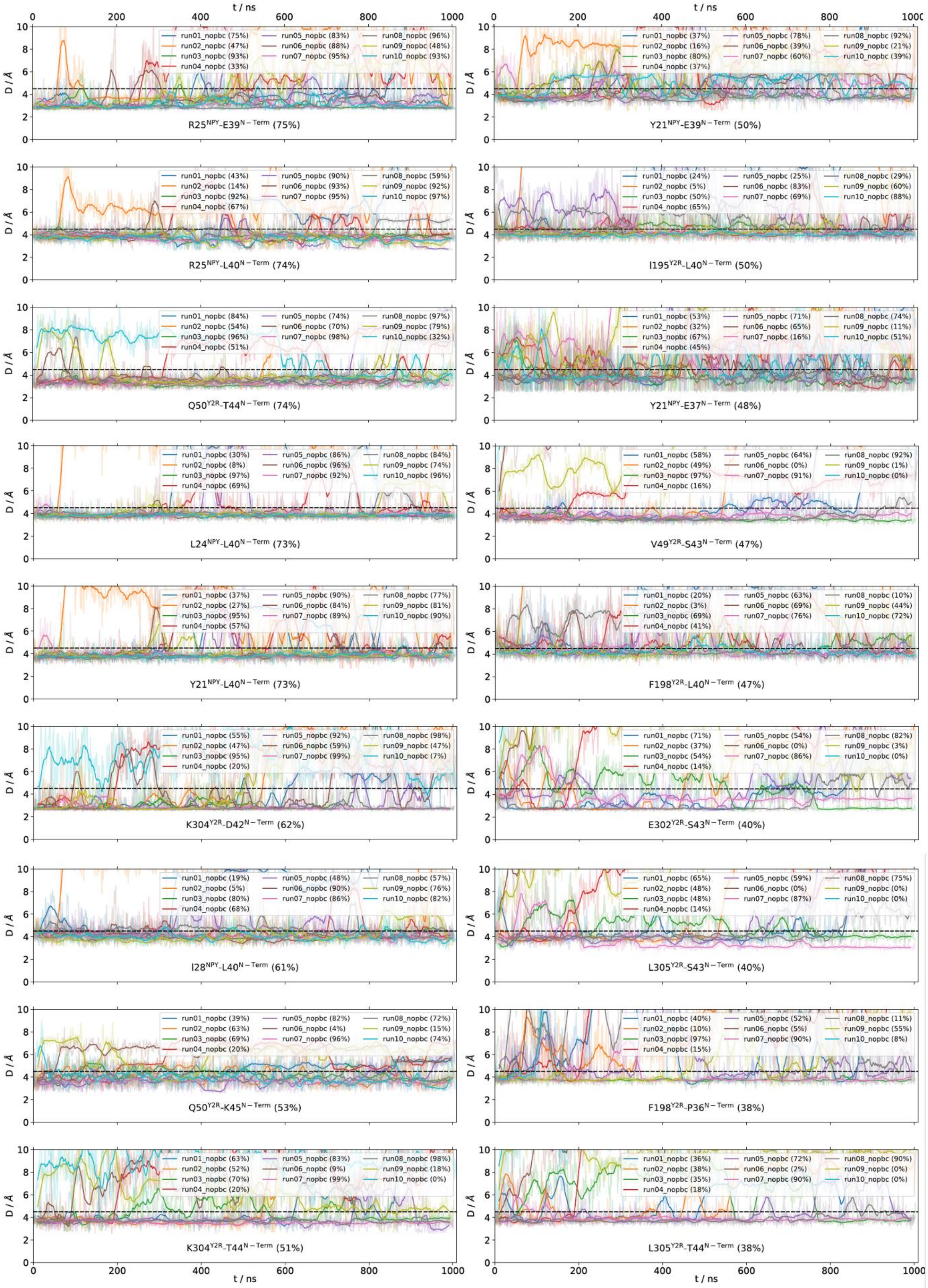

Supplementary Figure 7: Time traces of the NT interface contacts. The plots show the minimal distance over time between non-hydrogen atoms of the residue pairs indicated in each graph. Residues were considered to be in contact if the distance was equal to or less than 4.5

Å (indicated by the black dotted line). For better visibility, the time traces were smoothed by averaging over ten frames per point. The original, unsmoothed time trace is shown in the same color with higher transparency. Only residue pairs that were in contact for at least 38% of the overall simulation time are shown in this figure**.**

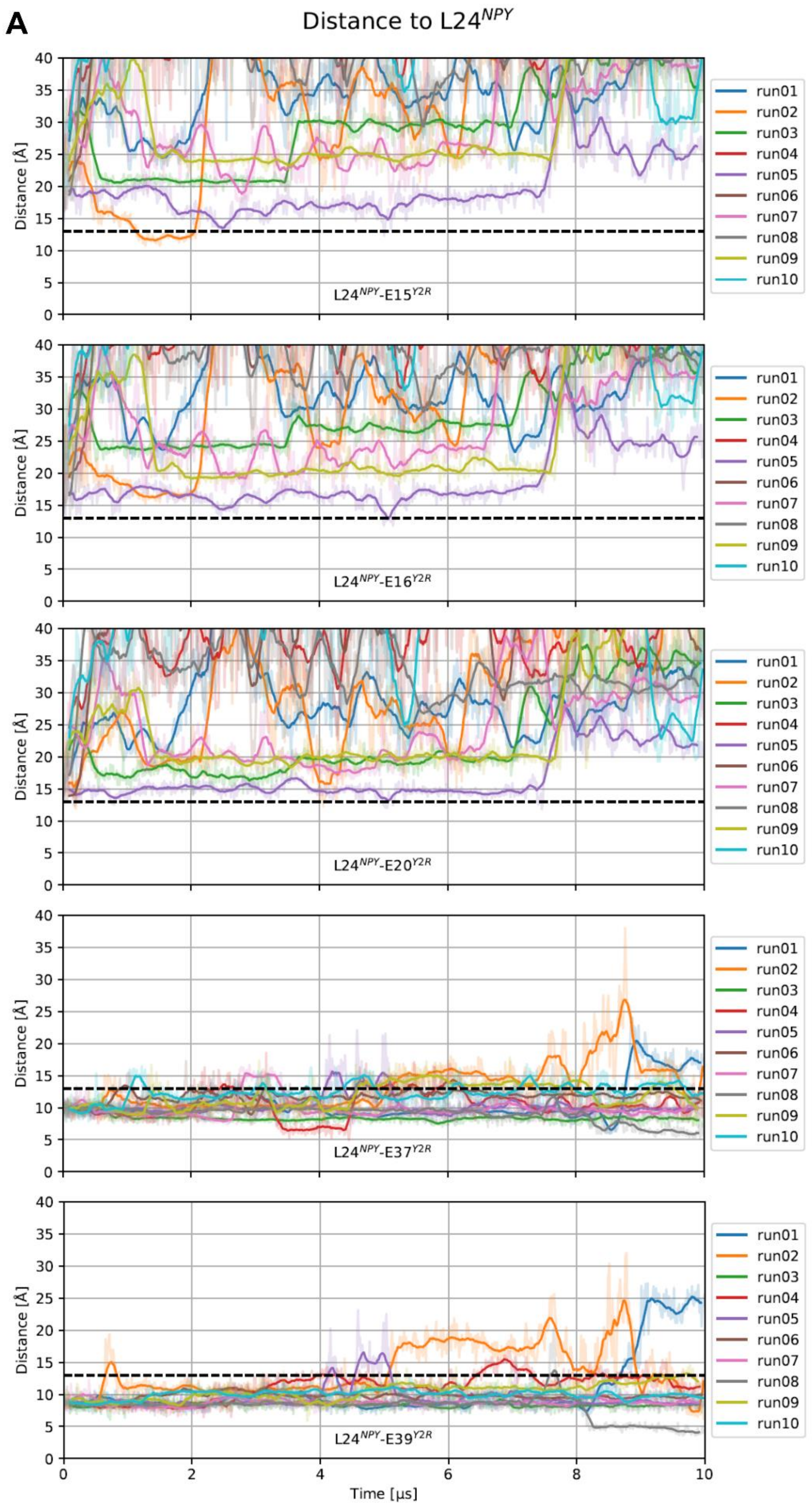
A
Distance to L24^{NPY}
L24^{NPY}-E15^{Y2R}
L24^{NPY}-E16^{Y2R}
L24^{NPY}-E20^{Y2R}
L24^{NPY}-E37^{Y2R}
L24^{NPY}-E39^{Y2R}
Distance [Å]
Time [µs]
run01
run02
run03
run04
run05
run06
run07
run08
run09
run10

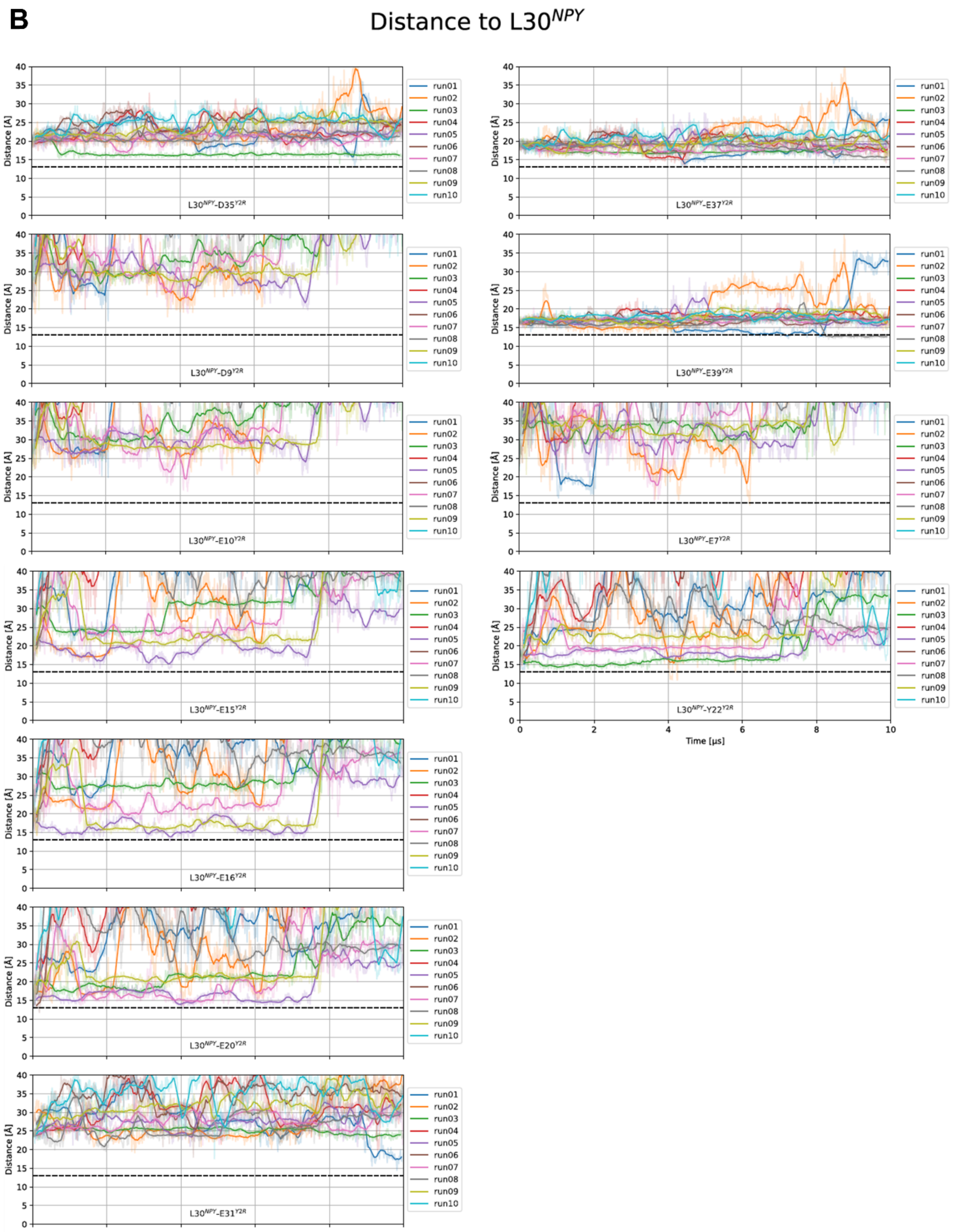

Supplementary Figure 8: C_β-C_β Distance plots between NPY residues L24 (A)/L30 (B) and Y_2R NT residues identified by the crosslinking experiment. The plots show the minimal distance over time between the C_β atoms of the residue pairs indicated in each graph. Residues were considered to be able to crosslink if the C_β-C_β distance ≤ 13 Å (indicated by the black dotted line). For better visibility, the time traces were smoothed by averaging over ten frames per point. The original, unsmoothed time trace is shown in the same color with higher transparency.

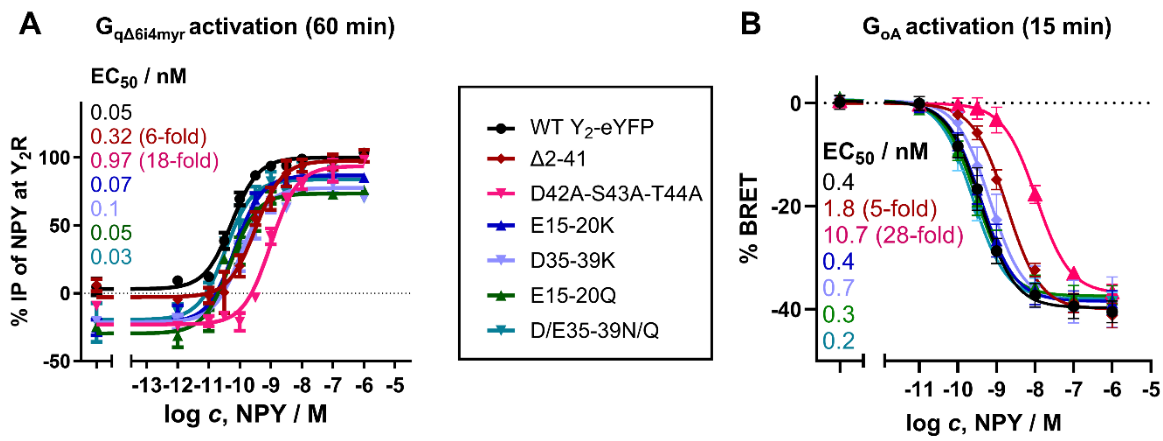

Supplementary Figure 9: G protein activation of Y_2R NT variants measured in alternative experimental settings provide highly similar outcomes. A) G protein activation was re-routed to the phospholipase C pathway by co-transfection of a chimeric $G_{iq4\Delta myr}$, and signal was read out by quantifying cellular inositol phosphate level after 60 min NPY stimulation. B) G protein activation measured by a G-CASE BRET sensor[100] after 15 min of NPY stimulation. Data are the mean ± SEM of n=3 (A), n=5-9 (B) independent experiments each performed in technical triplicate.

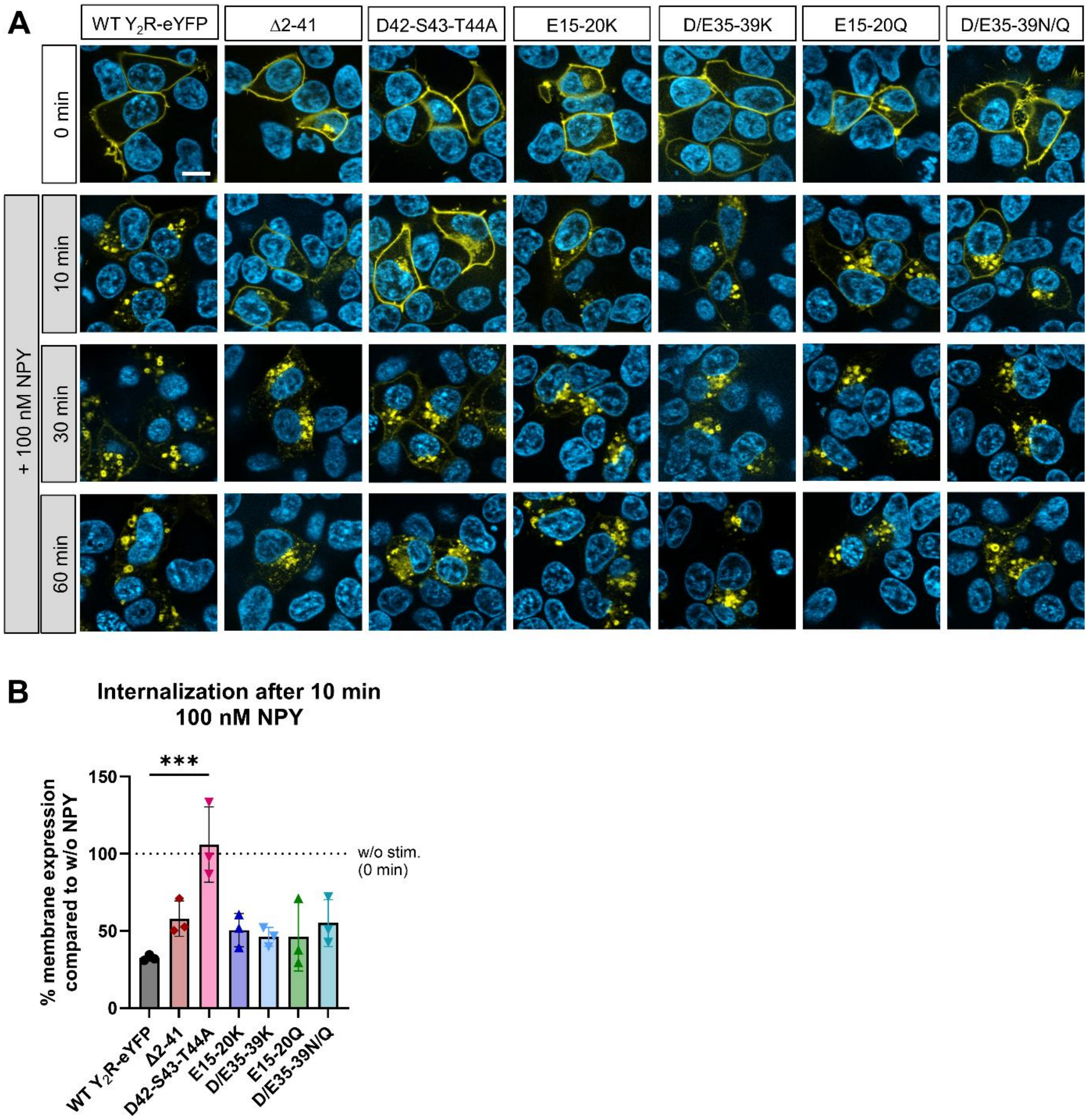

Supplementary Figure 10: NPY-induced internalization of Y_2R NT variants in live HEK293 cells. HEK293 cells were transiently transfected with Y_2R-eYFP variants (yellow) and imaged before and after stimulation with 100 nM NPY over 60 min. Nuclei were stained with H33342, scale bar equals 10 µm. A) While wild-type Y_2R internalized in endosomal structures already after 10 min ligand stimulation and the plasma membrane is hardly visible, Δ2-41 and D42A-S43A-T44A display delayed internalization in agreement with their weaker recruitment of arrestin-3. B) Quantification of membrane-bound receptors after 10 min stimulation with 100 nM NPY. Shown is normalized mean ± SEM from three independent experiments with each N=40 measurements per condition. 1-way-ANOVA with Dunnett's post-hoc test against wild-type Y_2R. *** $p < 0.001$.

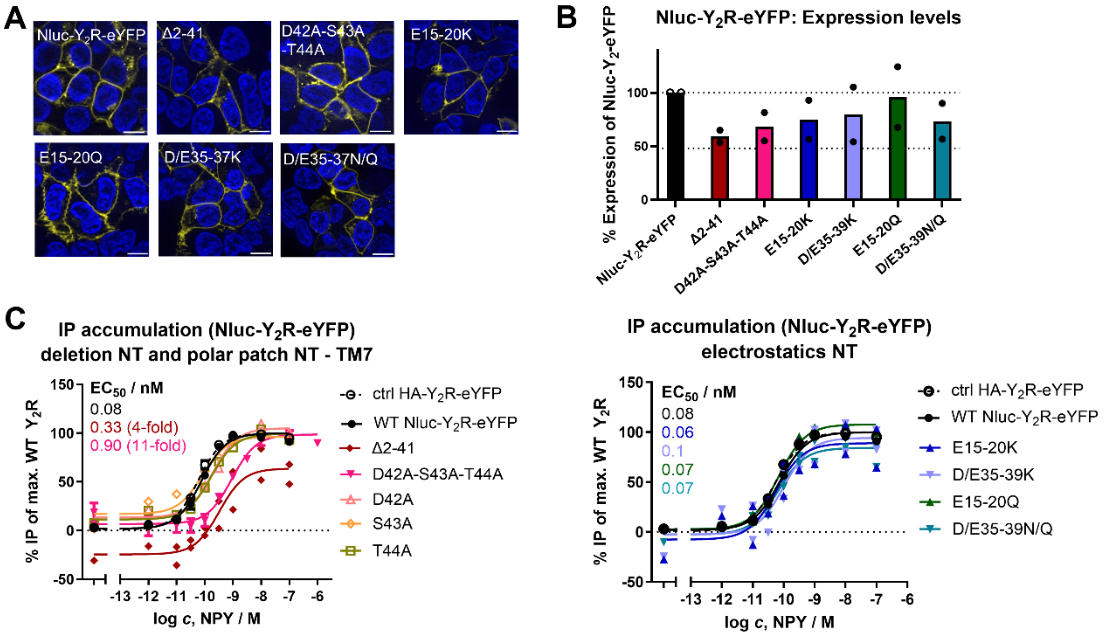

Supplementary Figure 11: Expression and activity of Nluc-Y_2R-eYFP variants used for NanoBRET binding. A) Live cell fluorescence microscopy shows that Nluc-tagged Y_2R NT variants are transported to the cell surface. Nluc-Y_2R-eYFP variants are shown in yellow, cell nuclei are stained by H33342 and shown in blue, scale bar equals 10 µm. All pictures were acquired with identical light exposure and picture processing. B) Quantification of cellular receptor expression based on eYFP fluorescence in a plate reader; data are the means and individual values of two independent experiments relative to Nluc-Y_2R-eYFP. C) Activity of Nluc-Y_2R variants towards G-protein activation measured by inositol phosphate accumulation. Chimeric $G_{iq4\Delta myr}$ was co-transfected to re-route signaling towards the phospholipase C pathway. The activity of Nluc-Y_2R-eYFP is virtually indistinguishable from HA-Y_2R-eYFP, and N-terminal variants appear wild-type-like with exception of D42A-S43A-T44A and Δ2-41. Data are the mean ± SEM or single replicates (Δ2-41; E15-20K, D/E35-39K; E15-20Q; D/E35-39N/Q) of n=2-5 independent experiments each performed in technical triplicate.

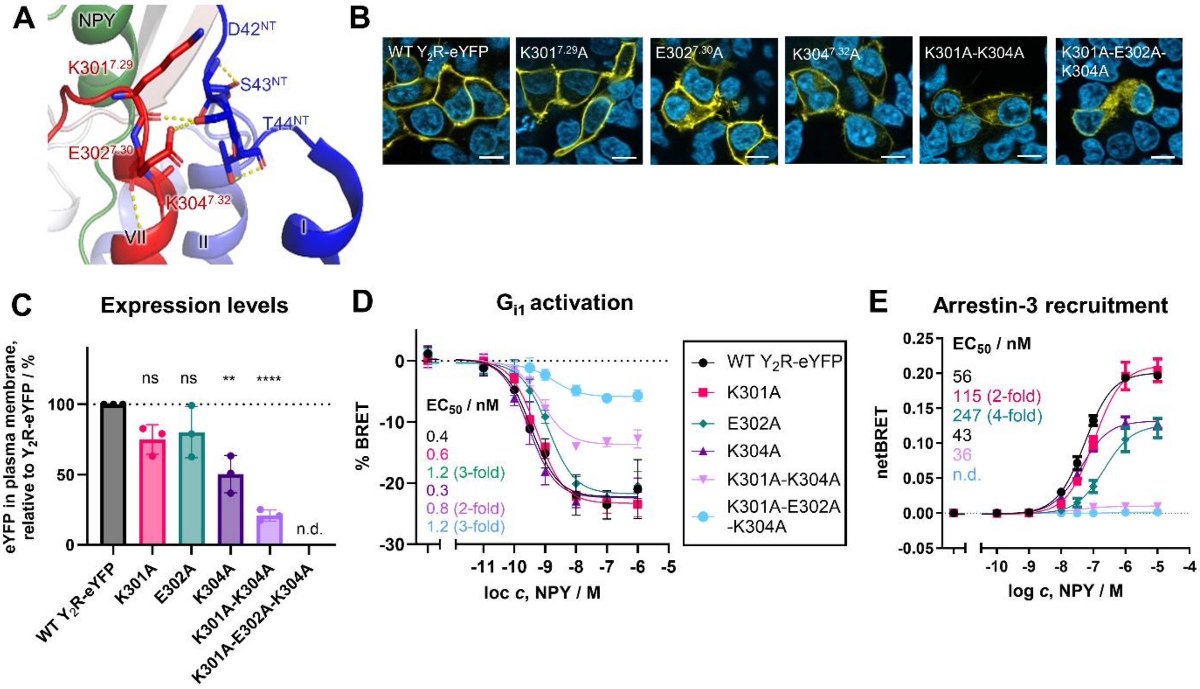

Supplementary Figure 12: Functional characterization of Y_2R ECL3 residues in contact with the membrane-proximal NT. A) Close-up view to interactions of ECL3 with membrane proximal Y_2R NT from the cryo-EM structure (PDB 7X9B) B) Live cell fluorescence microscopy of single and combination mutants in ECL3. While the single mutants largely show wild-type-like expression, the double mutant K301$^{7.29}$A-K304$^{7.32}$A has reduced plasma membrane expression, and the triple mutant K301$^{7.29}$A-E302$^{7.30}$A-K304$^{7.32}$A is retained intracellularly. Y_2R-eYFP variants are shown in yellow, cell nuclei are stained by H33342 and shown in blue, scale bar equals 10 µm. All pictures were acquired with identical light exposure and picture processing. C) Quantification of receptors in the plasma membrane based on microscopy experiments as shown in B from N≥40 cells in three independent experiments, relative to wild-type Y_2R. Plasma membrane expression was not determinable (n.d.) in the triple variant. 1-way-ANOVA with Dunnett's post-hoc test against wild-type Y2R. * $p<0.05$, ** $p< 0.01$, *** $p < 0.001$, **** $p<0.0001$. D) Activity of Y_2R variants towards G_{i1} proteins as measured by a direct BRET assay (G_{i1}-CASE) 10 min after ligand stimulation. E302$^{7.30}$A displays a three-fold shift in potency despite wild-type-like expression. Concentration-response of double mutant K301$^{7.29}$A-K304$^{7.32}$A and triple mutant K301$^{7.29}$A-E302$^{7.30}$A-K304$^{7.32}$A is likely affected by their very low membrane expression (*cf.* B and C). E) Recruitment of arrestin-3 to Y_2R variants as measured by BRET 10 min after NPY stimulation. E302$^{7.30}$A displays a four-fold shift in potency and 35% reduced $BRET_{max}$ despite wild-type-like expression. Concentration-response of double mutant K301$^{7.29}$A-K304$^{7.32}$A and triple mutant K301$^{7.29}$A-E302$^{7.30}$A-K304$^{7.32}$A is likely affected by their very low membrane expression (*cf.* B and C). Data in C–E are the mean ± SEM of n=3 independent experiments each performed in technical triplicate.

Supplementary Tables

Supplementary Table 1: Signaling of Y_2R NT variants. All values are given as mean (95% CI). Statistical significance in each signaling pathway (row) was tested in a one-way-ANOVA with Dunnett's post-hoc test corrected for multiple comparison against wild type Y_2R. * P<0.05, ** P<0.01, *** P<0.001, **** P<0.0001.

		**WT Y_2R-eYFP**	**Δ2-41**	**D42A-S43A-T44A**	**E15-20K**	**D/E35-39K**	**E15-20Q**	**D/E35-39N/Q**
G_{i1}	$\%BRET_{max}$	-23.0 (-23.7 to -22.2)	-22.4 (-23.6 to -21.2)	-21.7 (-22.9 to -20.5)	-23.3 (-24.5 to -22.1)	-22.3 (-23.2 to -21.4)	-21.6 (-22.8 to -20.5)	-21.6 (-22.7 to -20.5)
	$logEC_{50}$	-9.35 (-9.42 to -9.28)	-8.82 **** (-8.94 to -8.70)	-8.264 **** (-8.39 to -8.14)	-9.38 (-9.50 to -9.27)	-9.04 ** (-9.14 to -8.94)	-9.54 (-9.67 to -9.27)	-9.37 (-9.49 to -9.26)
	k_{obs} at 1 nM NPY / s^{-1}	0.0102 (0.0068 to 0.0137)	0.0230 (0.0085 to 0.0374)	0.0196 (0 to 0.0401)	0.0135 (0.0102 to 0.0168)	0.0110 (0.0055 to 0.0165)	0.0122 (0.0082 to 0.0162)	0.0107 (0.0066 to 0.0148)
G_{oA}	$\%BRET_{max}$	-39.7 (-40.7 to -38.8)	-40.0 (-40.9 to -39.0)	-36.9 (-38.1 to -35.8)	-38.5 (-39.6 to -37.3)	-38.6 (-40.5 to -36.8)	-37.5 (-38.6 to -36.3)	-38.0 (-38.9 to -37.2)
	$logEC_{50}$	-9.39 (-9.44 to -9.33)	-8.74 **** (-8.80 to -8.67)	-7.97 **** (-8.04 to -7.90)	-9.38 (-9.45 to -9.31)	-9.15 **** (-9.25 to -9.04)	-9.50 (-9.58 to -9.43)	-9.59 *** (-9.64 to -9.53)
G_{qi}	E_{max} / %	100 (96.3 to 103.6)	97.6 (90.3 to 104.8)	93.6 (84.5 to 102.6)	86.7 (83.4 to 90.0)	77.6 ** (70.0 to 85.1)	73.4 *** (66.4 to 80.3)	83.8 * (75.9 to 91.7)
	$logEC_{50}$	-10.27 (-10.39 to -10.15)	-9.50 *** (-9.71 to -9.29)	-9.01 **** (-9.21 to -8.82)	-10.16 (-10.25 to -10.06)	-9.98 (-10.20 to -9.76)	-10.30 (-10.52 to -10.09)	-10.46 (-10.71 to -10.21)
Arr3	$BRET_{max}$	0.201 (0.194 to 0.209)	0.150 *** (0.143 to 0.156)	0.088 **** (0.074 to 0.103)	0.155 *** (0.143 to 0.168)	0.099 **** (0.091 to 0.108)	0.236 ** (0.222 to 0.250)	0.218 (0.199 to 0.237)
	$logEC_{50}$	-7.26 (-7.34 to -7.19)	-6.54 **** (-6.62 to -6.45)	-6.01 **** (-6.27 to -5.74)	-7.35 (-7.50 to -7.20)	-7.23 (-7.40 to -7.05)	-7.37 (-7.48 to -7.25)	-7.42 (-7.59 to -7.24)
	k_{obs} at 100 nM NPY / s^{-1}	0.0060 (0.0042 to 0.0078)	0.0106 * (0.0053 to 0.0159)	0.0085 *at 1 µM NPY* (0.0006 to 0.0164)	0.0120 ** (0.0087 to 0.0152)	0.0114 ** (0.0098 to 0.0130)	0.0089 (0.0064 to 0.0114)	0.0092 (0.0053 to 0.0131)

Supplementary Table 2: Binding properties of Y_2R NT variants. All values are given as mean (95% CI). The K_D, high of D42A-S43A-T44A has a very wide 95% CI and is therefore shown in *italics*. Statistical significance in each signaling pathway (row) was tested in a one-way-ANOVA with Dunnett's post-hoc test corrected for multiple comparison against wild type Y_2R. * P<0.05, ** P<0.01, *** P<0.001., **** P<0.0001. n.d. not determinable due to small measuring window.

	**WT Nluc-Y_2R-eYFP**	**Δ2-41**	**D42A-S43A-T44A**	**E15-20K**	**D/E35-39K**
$BRET_{max}$	0.0292 (0.0273 to 0.0311)	0.6520 **** (0.5907 to 0.7133)	0.0060 (0.0040 to 0.0080)	0.0307 (0.0243 to 0.0371)	0.0270 (0.025 to 0.0287)
Fraction high affinity	0.0617 (0.0352 to 0.0881)	0.0412 (0.0357 to 0.0467)	0.1507 ** (0.0649 to 0.2365)	0.0783 (0.0705 to 0.0862)	0.0957 (0 to 0.1938)
Log $K_{D,\ high}$	-9.57 (-10.25 to -8.90)	-9.64 (-9.94 to -9.33)	*-10.20* (-13.50 to -6.90)	-9.46 (-10.48 to -8.44)	-9.65 (-10.64 to -8.66)
[$K_{D,\ high}$ replicates]	-9.88, -9.40, -9.43	-9.51, -9.76, -9.64	-9.81, -11.68, -9.11	-9.24, -9.21, -9.93	-9.38, -9.46, -10.11
Log $K_{D,\ low}$	-6.54 (-6.59 to -6.49)	-6.18 *** (-6.24 to -6.12)	-6.12 *** (-6.47 to -5.77)	-6.56 (-6.65 to -6.47)	-6.62 (-6.80 to -6.44)
[$K_{D,\ low}$ replicates]	-6.52, -6.56, -6.54	-6.16, -6.18, -6.21	-6.11, -6.26, -5.98	-6.58, -6.52, -6.58	-6.53, -6.67, -6.65
k_{off}, fast / s^{-1}	0.052 (0.009 to 0.095)	0.149 * (0.027 to 0.272)	n.d.	0.123 (0.078 to 0.168)	0.105 (0.054 to 0.156)

Supplementary Table 3: Signaling of Y_2R ECL3 variants. All values are given as mean (95% CI). Statistical significance in each signaling pathway (row) was tested in a 1-way-ANOVA with Dunnett's post-hoc test corrected for multiple comparison against wild type Y_2R. * P<0.05, ** P<0.01, *** P<0.001, ****P<0.0001. n.d. not determinable due to small measuring window.

		**WT Y_2R-eYFP**	**K301A**	**K304A**	**K301A-K304A**	**E302A**	**K301A-E302A-K304A**
G_{i1}	$\%BRET_{max}$	-22.3 (-23.9 to -20.6)	-23.3 (-24.2 to -22.5)	-22.4 (-23.7 to -21.1)	-13.7 **** (-14.5 to -12.8)	-21.7 (-23.1 to -20.4)	-5.8 **** (-6.7 to -4.9)
	$logEC_{50}$	-9.44 (-9.62 to -9.26)	-9.25 (-9.33 to -9.17)	-9.51 (-9.65 to -9.38)	-9.11 (-9.25 to -8.97)	-8.93 ** (-9.08 to -8.78)	-8.92 ** (-9.32 to -8.49)
G_{oA}	$\%BRET_{max}$	-37.4 (-39.0 to -35.9)	-43.2 ** (-44.4 to -42.0)	-33.9 * (-37.0 to -30.9)	-19.1 **** (-20.7 to -17.6)	-36.0 (-37.0 to -35.0)	-5.4 **** (-6.4 to -4.6)
	$logEC_{50}$	-9.42 (-9.52 to -9.33)	-9.38 (-9.44 to -9.31)	-9.51 (-9.73 to -9.30)	-8.88 ** (-9.08 to -8.68)	-8.60 *** (-8.67 to -8.53)	-7.74 **** (-8.11 to -7.37)
Arr3	$BRET_{max}$	0.203 (0.195 to 0.210)	0.215 (0.200 to 0.230)	0.133 **** (0.125 to 0.140)	0.010 **** (0.009 to 0.012)	0.131 **** (0.115 to 0.152)	0.002 **** (0.001 to ???)
	$logEC_{50}$	-7.25 (-7.33 to -7.18)	-6.94 (-7.07 to -6.81)	-7.37 (-7.47 to -7.26)	-7.45 (-7.76 to -7.16)	-6.61 ** (-6.85 to -6.33)	nd

Supplementary Table 4. Rosetta distance constraints between the Y_2 receptor (chain A) and the NPY peptide (chain B).

Constraint Type	Atom Type	Residue Number and chain ID (Receptor)	Atom Type	Residue Number and chain ID (NPY)	Constraint Function	Target Distance	Force Constant	Flatness
AtomPair	CB	20A	CB	24B	FLAT_ HARMONIC	13	1	2
AtomPair	CB	39A	CB	24B	FLAT_ HARMONIC	13	1	2
AtomPair	CB	16A	CB	30B	FLAT_ HARMONIC	13	1	2
AtomPair	CB	20A	CB	30B	FLAT_ HARMONIC	13	1	2
AtomPair	CB	37A	CB	30B	FLAT_ HARMONIC	13	1	2
AtomPair	CB	39A	CB	30B	FLAT_ HARMONIC	13	1	2
AtomPair	CB	211A	CB	30B	FLAT_ HARMONIC	13	1	2

Supplementary Table 5: Description of the properties of the simulation box used for the MD simulation.

**MD Setup properties**	**Setup**
Simulation Box Dimensions (x*y*z)	160.11 Å * 160.11 Å * 163.256 Å
Total number of atoms	394188
Total number of water molecules (TIP3)	97452
Total number of salt molecules	0 Na^{+}, 2 Cl^{-}

Supplementary Table 6: Equilibration steps used to adjust the Y_2R model for the charmm36 forcefield. BB restraints: flexible, positional restraints of protein backbone; SC restraints: flexible, positional restraints of protein side chains; Lipid restraints: flexible, positional restraints for POPC molecules.

**Step**	**step 1**	**step 2**	**step 3**	**step 4**	**step 5**	**step 6**	**step 7**
**Duration**	0.125 ns	0.125 ns	0.125 ns	0.5 ns	100 ns	125 ns	90 ns
**BB restraint**	4000	2000	1000	500	200	50	25
**SC restraint**	2000	1000	500	200	50	0	0
**Lipid restraints**	1000	400	400	200	40	0	0

Supplementary Table 7: Primers used to create Y_2R mutants

**Name**	**Sequence (5'-3')**
Y2-D42A_for	GAGCTTATAGCTAGTACCAAGCTGATTGAGGTACAAGTTGTTCTCATATTG
Y2-D42A_rev	GGTACTAGCTATAAGCTCTGGCTCAGGGTCAGGGACC
Y2-S43A_for	CTTATAGATGCTACCAAGCTGATTGAGGTACAAGTTGTTCTCATATTG
Y2-S43A_rev	CTTGGTAGCATCTATAAGCTCTGGCTCAGGGTCAGGGACC
Y2-T44A_for	TAGATAGTGCCAAGCTGATTGAGGTACAAGTTGTTCTCATATTGGC
Y2-T44A_rev	ATCAGCTTGGCACTATCTATAAGCTCTGGCTCAGGGTCAGGGAC
Y2-K45A_for	GATAGTACCGCCCTGATTGAGGTACAAGTTGTTCTCATATTGGCCTAC
Y2-K45A_rev	CTCAATCAGGGCGGTACTATCTATAAGCTCTGGCTCAGGGTCAGGGAC
Y2-D42A-S43A-T44A_for	GCTGCTGCCAAGCTGATTGAGGTACAAGTTGTTCTCATATTGGCCTAC
Y2-D42A-S43A-T44A_rev	CTCAATCAGCTTGGCAGCAGCTATAAGCTCTGGCTCAGGGTCAGG
Y2-Del-2-41_for	GATAGTACCAAGCTGATTGAGGTACAAGTTGTTCTC
Y2-Del-2-41_rev-Nluc	CAATCAGCTTGGTACTATCACCGCTGCCTCCGCCTCCGCTCGC
Y2-Del-2-41_rev-wo	CAATCAGCTTGGTACTATCCATGGTGGCCTTGAGCTCGACAATCG
Y2-E15-16-20-K_for	CAGTGAAAAAGATGAAGGTGAAACAATACGGGCCACAAACAACTCC
Y2-E15-16-20-K_rev	GTATTGTTTCACCTTCATCTTTTTCACTGTCTGGTTCTCATCAGCCTCTGC
Y2-D35-E37-E39-K_for	CCTAAACCTAAGCCAAAGCTTATAGATAGTACCAAGCTGATTGAGG
Y2-D35_E37_E39-K_rev	ATAAGCTTTGGCTTAGGTTTAGGGACCAGTTCACCTCTAGGAGTTGTTTG
Y2-E15-16-20Q_for	CAGTGCAACAAATGAAGGTGCAGCAATACGGGCCACAAACAACTCC
Y2-E15-16-20_Q_rev	GTATTGCTGCACCTTCATTTGTTGCACTGTCTGGTTCTCATCAGCCTCTGC
Y2-D35N-E37Q-E39Q_for	CCTAACCCTCAGCCACAGCTTATAGATAGTACCAAGCTGATTGAGG
Y2-D35N-E37Q-E39Q_rev	ATAAGCTGTGGCTGAGGGTTAGGGACCAGTTCACCTCTAGGAGTTGTTTG

Supplementary Table 8: PASEF DIA isolation window

```
#MS Type,Cycle Id,Start IM [1/K0],End IM [1/K0],Start Mass [m/z],End Mass [m/z],CE [eV]
MS1,0,-,-,-,-,-
PASEF,1,0.9609,1.4398,1071.00,1171.00,-
PASEF,1,0.6500,0.9600,385.00,485.00,-
PASEF,2,1.0497,1.5769,1267.00,1367.00,-
PASEF,2,0.6944,1.0285,483.00,583.00,-
PASEF,3,0.7388,1.0971,581.00,681.00,-
PASEF,4,0.7832,1.1656,679.00,779.00,-
PASEF,5,0.8277,1.2342,777.00,877.00,-
PASEF,6,0.8721,1.3027,875.00,975.00,-
PASEF,7,0.9165,1.3713,973.00,1073.00,-
PASEF,8,1.0053,1.5084,1169.00,1269.00,-
```

Supplementary Methods

More detailed description of Cross-linking, data acquisition and data analysis

Cross-linking

Aliquots (100 µL) containing 1.37 µM bicelle-refolded Y_2R or N-terminal peptide of Y_2R (NT-Y_2R) solution in 50 mM aqueous 2-[4-(2-hydroxyethyl)-piperazine-1-yl]ethanesulfonic acid (HEPES), pH 7.2, and 1.5 mM 1,2- diheptanoyl-sn-glycero-3-phosphocholine (DHPC) were incubated for 1 hour on ice and darkness with a triply diazirine-substituted NPY variant (final concentration of 13.6 µM). Cross-linking was induced by UV-A irradiation with a LED lamp (ANUJ6186, Panasonic) for 30 s for a total of 30 joules/cm^2. For each cross-linking system triplicate cross-linking reactions were prepared and one negative control (NC) where no NPY was added; 3 x NPY-Y_2R + 1 x Y_2R NC and 3 x NPY-NT-Y_2R + NT-Y_2R NC. All samples were prepared using Suspension-Trapping (S-Trap™; Protifi) assisted protein digestion. Each replicate was mixed with 100 µL solution of 10% (w/v) sodium dodecyl sulfate (SDS) in 100 mM aqueous tris(hydroxymethyl)aminomethane hydrochloride (Tris-HCl), pH 7.5. The samples were reduced by adding 8.7 µL of a Tris(2-carboxyethyl)phosphine hydrochloride (TCEP) stock solution (120 mM in deionized water (dH_2O), final concentration of 120 mM) and incubating at 55 °C for 15 min. Reduced cysteines were alkylated by adding 8.7 µL of iodoacetamide (IAA) stock solution (500 mM in dH_2O, final concentration of 20 mM) and allowing reaction to proceed for 10 min at room temperature (RT) in darkness. The solution was acidified with 21.7 µL of aqueous 27.5% (v/v) phosphoric acid (final concentration of ~2.5%) and vortexed. Prior to loading into S-Trap columns, each replicate was mixed with 1435 µL of binding/washing solution (100 mM Tris-HCl, pH 7.5, in 90% (v/v) Methanol). Samples were loaded sequentially in 150 µL aliquots and centrifuged at 4,000 x g for 30 s until all sample was transferred to the S-Trap columns. Additional 150 µL of binding/washing solution were added and passed-through by centrifugation at 4,000 x g for 30 s three times. After the last washing step, the S-Trap columns were centrifuged again at 4,0000 x g for 1 min. Then, each S-Trap column was transferred to a clean Eppendorf Protein LoBind 2 mL tubes. To the top of each S-Trap column, 1 µg of AspN dissolved in 20 µL of aqueous 50 mM ammonium bicarbonate (ABC) was added to reach a 100:1 (protein:enzyme) ratio. The samples were transferred to a wet chamber at 37 °C and allowed the digestion to proceed overnight. Second enzymatic digestion was performed by adding 4 µg of Trypsin dissolved in 20 µL of aqueous 50 mM ABC to reach a 25:1 (protein:enzyme) ratio. After second digestion proceeded for 14 h, 40 µL of 50 mM ABC solution was added to each sample and centrifuged at 4,000 x g for 1 min. Afterwards, 40 µL of aqueous 0.2% (v/v) formic acid were added and centrifuged at 4,000 x g for 1 min. Last elution step was performed by adding 40 µL of aqueous 50% (v/v) acetonitrile (ACN) and centrifugation at 4,000 x g for 1 min. The peptide solutions were dried under vacuum and stored at -20 °C.

LC-TIMS-MS/MS Data Collection

Dried peptides were reconstituted by first adding with 5 µL of aqueous 30% (v/v) ACN with 0.05% (v/v) trifluoroacetic acid (TFA), vortexing and short-spinned. The mixture was diluted with 45 µL of aqueous 0.05% TFA before injections to a final ACN concentration of 3% (v/v). For each analysis, 20% of the total sample was loaded on column corresponding to an estimated 20 µg of digest (based on initial protein content). Peptides were separated on an UltiMate 3000 RSLC nano-HPLC system (Thermo Fisher Scientific) that was coupled to a timsTOF Pro mass spectrometer (Bruker Daltonics). Peptides were trapped on a C18 column

(precolumn Acclaim PepMap 100, 300 µm × 5 mm, 5 µm, 100 Å) (Thermo Fisher Scientific) and separated on a µPAC 50 column (PharmaFluidics) or a self-packed Picofrit (New Objective) nanospray emitter (360 µm ID × 75 µm ID x 150 mm L, 15 µm Tip ID) packed with a C18-stationary phase (3.0 µm, 120 Å) (Dr. Maisch GmbH). After sample load, the precolumn was washed for 15 minutes with aqueous 0.1 % (v/v) TFA at a flow rate of 30 µl/min and a pre-column temperature 50°C. Peptide elution and separation on the µPAC column was performed with a linear 90 min water−acetonitrile (ACN) gradient from 3% to 50% B where A is aqueous 0.1% (v/v) formic acid and B is 0.1% (v/v) formic acid in ACN. Additionally, a flow gradient was employed ranging from 900 to 600 nl/min. The column was washed at a flow rate of 600 nL/min with the following gradient: 50% to 85% B ACN (5 min), 85% B (5 min), 85% B to 3% B ACN (5 min), 3% B (15 min). Only cross-linking samples generated from the bicelles embedded with Y_2R (Y_2R-bicelle) were analyzed with the µPAC column. All subsequent samples were analyzed with the self-packed C18 column. On the self-packed C18 column, peptides were eluted and separated using a linear gradient from 3% to 50% B (with solvent B: 0.1% (v/v) formic acid in ACN) with a constant flow rate of 300 nl/min over 90 min, 50% to 85% B (1 min) and 85% B (9 min). The separation column is kept at 40°C using an external column heater (Sonation GmbH). To align results obtained from different chromatographic conditions, unmodified peptides identified in all samples were used as endogenous indexed retention time (iRT) standards to create a retention time calculator to account for retention time shifts.

After chromatographic separation the peptides were ionized through electrospray ionization (ESI) with a capillary voltage of 1500 V and facilitated drying with N_2 gas at 180 °C and flow rate of 3.0 L/min. The generated ions were then analyzed by TIMS in a dual trapped ion mobility cell setup prior to tandem mass spectrometry (MS/MS) detection. The TIMS-MS/MS data were acquired both in DDA-PASEF and DIA-PASEF mode. Ion accumulation and ramp time was performed in 200 ms. Two mobility-dependent collision energy ramps were used: 1) 59 eV at an inversed reduced mobility ($1/K_0$) of 1.6 $V{\cdot}s/cm^2$ and 20 eV at 0.60 $V{\cdot}s/cm^2$ and 2) 95 eV at 1.60 $V{\cdot}s/cm^2$ and 20 eV at 0.60 $V{\cdot}s/cm^2$. Collision energies were linearly interpolated between these two $1/K_0$ values and kept constant above or below the set values, respectively. For DDA-PASEF experiments, the target intensity per individual PASEF precursor was set to 100 000 with an intensity threshold of 1000. 10 PASEF MS/MS ramps were triggered per acquisition cycle (2.47 s). Precursor ions in an *m/z* range between 100 and 1700 with charge states ≥2+ and ≤8+ were selected for fragmentation. Active exclusion was enabled for 0.5 min (mass width 0.015 Th, $1/K_0$ width 0.100 $V{\cdot}s/cm^2$) with early re-targeting if the precursor intensity had a 4x improvement in precursor intensity. For DIA-PASEF, isolation schemes were adapted to the sample matrix based on cross-linked peptides identified in the DDA-PASEF data. The DIA-PASEF isolation scheme defined with timsControl (v 3.0.21) *DIA-PASEF Window Editor* function is provided in Supplementary Table 8. After acquisition, DDA-PASEF data was processed with DataAnalysis (v5.3; Bruker Daltonics) to generate fragment ion spectra peak lists in mascot generic format text files (i.e., *.mgf*). Collected fragment ion spectra were combined if their precursor ions were collected within a 0.75 min window, the monoisotopic *m/z* of the targeted precursors was within 0.015 *m/z*, and the precursors $1/K_0$ were within 0.025 $V{\cdot}s{\cdot}cm^{-2}$.

Data Validation and Processing

<u>Cross-linked peptides identification</u>

Identification of cross-links was performed with MeroX (v. 2.0.1.7) (Götze et al., 2019) from the *.mgf* files generated with DataAnalysis. The annotation of Y_2R-NPY cross-links was performed with the following settings: semi-specific proteolytic cleavage: C-terminal at Lys and Arg

(trypsin) and N-terminal at Asp and Glu (Asp-N) with up to 3 missed cleavages for each and a maximum of 5 total missed cleavages; peptide lengths of 4 to 25 amino acids; PTMs: alkylation of Cys by iodoacetamide (fixed), oxidation of Met; an artificial amino acid was defined in MeroX as "*I*" to consider acid (2*S*)-2-Amino-3-(3-methyl-3*H*-diazirin-3-yl)propanoic acid (photo-leucine; pLeu) with an elemental composition of $C_6H_{11}NO$ and, for downstream analysis with proXL and Skyline, the cross-linker was defined as the mass shift with respect to Leucine (-CH_4); cross-linker specificity: site 1 was set to consider only NPY peptides containing "*I*" (i.e. pLeu) and site 2 was set to Asp, Glu, and the C- terminus for search set considering only acidic sites reactivity and site 2 was set to all 20 proteinogenic amino acids for search set considering reactivity to all amino acids; cross-link specific fragments (XL-fragments): evidence of two XL-fragments (Pep-N2: -CH_4 and Pep-N2+H2O: +O – CH_2) at site 1 were considered as optional and at site 2 XL-fragments where the whole cross-linker was missing (i.e., Δmass = 0) was considered as optional; search algorithm: Quadratic mode with a minimal peptide score of ≥0; a- , b-, and y-ion series were considered; precursor mass accuracy: 10 ppm; fragment ion mass accuracy: 10 ppm; 10% intensity as prescore cut-off; 1% false discovery rate (FDR) cut-off, and minimum score cut-off: 0.

MeroX results from each search set were combined and the combined results were exported as .csv files. Using an in-house written R script, a peptide and precursor-specific ion mobility library was extracted from grouped MeroX results. For each of the matched precursor ions, the mean $1/K_0$ and range were calculated from all the $1/K_0$ reported for all cross-link spectra matches (XSMs). These values were formatted into a Skyline ion mobility spectral library and exported as .csv files.

Non-crosslinked peptides identification

The same reformatted .mgf files used for XL-peptides identification were used for the identification of non-cross-linked peptides. Peak lists obtained from MS/MS spectra were identified using MS-GF+ version Release (v 2023.01.12) (Kim & Pevzner, 2014) and MyriMatch (v 2.2.140) (Tabb et al., 2007).The search was conducted using SearchGUI version (v 4.2.14) (Barsnes & Vaudel, 2018). Protein identification was conducted against a concatenated target/decoy protein database containing the mature version of porcine NPY (UniProt ID: P01304), the in-house produced cysteine deficient Y_2R, porcine trypsin (UniProt ID: P00761), flavastacin (UniProt ID: Q47899), and their respective reverse decoy sequences generated in SearchGUI. The identification settings were as follows: Trypsin, Semi-Specific, with a maximum of 2 missed cleavages 10.0 ppm as MS1 and 10.0 ppm as MS2 tolerances; fixed modifications: alkylation of Cys (+57.021464 Da), variable modifications: Oxidation of M (+15.994915 Da). Peptides and proteins were inferred from the spectrum identification results using PeptideShaker version 2.2.25 (Vaudel et al., 2015). Peptide Spectrum Matches (PSMs), peptides and proteins were validated at a 1.0% False Discovery Rate (FDR) estimated using the decoy hit distribution. All validation thresholds and algorithms specific settings are listed in the "SearchEngine parameters" and, together with the PeptideShaker project and .mzID results, can be obtained in the deposited ProteomeXChange data PDX051865 (https://proteomecentral.proteomexchange.org/cgi/GetDataset?ID=PXD051865).

Peptide ID import into Skyline and validation

Detailed analysis pipeline has been reported recently[43], but here we describe a summary of the validation procedure. MeroX results were converted with proXL (https://github.com/yeastrc/proxl-web-app). The .pep.xml output in combination with the .mgf

files were used to generate XL-peptides spectral libraries in Skyline with a maximal 0.05 q-value cutoff. Spectral libraries for unmodified peptides were generated using PeptideShaker's results using a Peptide confidence score of 0.99. Unmodified peptides from Y2R and the enzymatic background that could be detected in all replicates were chosen as endogenous iRT standards. All peptides annotated to Y_2R and cross-linked peptides were imported into the Skyline document. The parsed ion mobility library was added to the document prior to generating extracted ion chromatograms (EICs) of the first three isotopes of each precursor ion from raw DDA-PASEF files; a TOF resolving power of 60,000 and ion mobility resolving power of 40 were used. Only XL-peptides that had passed the initial manual annotation in MeroX, were detected in all sample preparation replicates, and did not show similar peptide chromatographic features in the negative control samples were kept as true XL-peptides. These peptides, with their curated retention time integration windows, were then indexed into a retention time calculator within Skyline using endogenous iRT standards as reference points. The iRTs of these XL-peptides, the ion mobility library generated from DDA-PASEF data, and the top-10 fragment ions (intensity-wise) of each precursor ion were then used to guide generation of EICs from raw DIA-PASEF data in Skyline.
