# Supplementary material for: Transient ligand contacts of the intrinsically disordered N-terminus of neuropeptide Y2 receptor regulate arrestin-3 recruitment
Source: Nat Commun. 2025 Sep 19;16:8326. doi: 10.1038/s41467-025-64051-4 (PMC12449476; doi:10.1038/s41467-025-64051-4)
Supplement: Supplementary file 2 — Description of Additional Supplementary Files [file 41467_2025_64051_MOESM2_ESM.pdf]

Description of Additional Supplementary Files

File name: Supplementary Data 1
Description: Overview of cross-linked peptides.

Column A: region in Y2R in which cross-link is detected
Column B: indicates if a fragment has been detected in the cross-linking experiment using full length Y2R folded into lipid bicelles
Column C: indicates if a fragment has been detected in the cross-linking experiment using a synthetic peptide covering the Y2R NT sequence
Column D: cross-linked peptide (sequence); @ shows exact cross-linked position e.g., @ 7 (position 7 of DENQTVE), 5 (position 5 of YYSApLR) one-letter amino acid code, pL= photoleucine
Column E: cross-linked amino acid in Y2R
Column F: position of photo-leucine (pL) in NPY
Column G: score MeroX software. Please see methods for assignment and Götze, M (2019, Anal Chem)
Column H: mass-to-charge ratio of XL peptides
Column I: charge of XL peptides
Column J: mass of the found protonated molecule ion, in atomic mass units
Column K: calculated mass, in atomic mass units
Column L: retention time of cross-linked peptide in chromatogram
Column M: deviation between found and calculated mass in parts per million (ppm)
Column N: sequence of found fragment 1 in one letter code
Column P/Q: start/end position of found peptide fragment 1 within Y2R or NPY
Column R: sequence of found fragment 2 in one letter code
Column T/U: start/end position of found peptide fragment 2 within Y2R or NPY
Column V/W: cross-linked position within peptide fragment 1/2
Column X: automated file output by MeroX allows assignment of the spectrum (and file path) of the cross-linked peptide; Scan of the cross-linked peptide; name of the file; Scan nummer; precursor collision energy in eV, retention time in minutes; ion mobility
