# Supplementary material for: Transient ligand contacts of the intrinsically disordered N-terminus of neuropeptide Y2 receptor regulate arrestin-3 recruitment
Source: Nat Commun. 2025 Sep 19;16:8326. doi: 10.1038/s41467-025-64051-4 (PMC12449476; doi:10.1038/s41467-025-64051-4)
Supplement: Supplementary file 4 — Reporting Summary [file 41467_2025_64051_MOESM4_ESM.pdf]

nature portfolio

Corresponding author(s): Anette Kaiser, Andrea Sinz

Last updated by author(s): Aug 14, 2025

# Reporting Summary

Nature Portfolio wishes to improve the reproducibility of the work that we publish. This form provides structure for consistency and transparency in reporting. For further information on Nature Portfolio policies, see our Editorial Policies and the Editorial Policy Checklist.

## Statistics

For all statistical analyses, confirm that the following items are present in the figure legend, table legend, main text, or Methods section.

| n/a | Confirmed | |
|---|---|---|
| | ☒ | The exact sample size (*n*) for each experimental group/condition, given as a discrete number and unit of measurement |
| | ☒ | A statement on whether measurements were taken from distinct samples or whether the same sample was measured repeatedly |
| | ☒ | The statistical test(s) used AND whether they are one- or two-sided<br>*Only common tests should be described solely by name; describe more complex techniques in the Methods section.* |
| ☒ | | A description of all covariates tested |
| | ☒ | A description of any assumptions or corrections, such as tests of normality and adjustment for multiple comparisons |
| | ☒ | A full description of the statistical parameters including central tendency (e.g. means) or other basic estimates (e.g. regression coefficient) AND variation (e.g. standard deviation) or associated estimates of uncertainty (e.g. confidence intervals) |
| ☒ | | For null hypothesis testing, the test statistic (e.g. *F*, *t*, *r*) with confidence intervals, effect sizes, degrees of freedom and *P* value noted<br>*Give P values as exact values whenever suitable.* |
| ☒ | | For Bayesian analysis, information on the choice of priors and Markov chain Monte Carlo settings |
| ☒ | | For hierarchical and complex designs, identification of the appropriate level for tests and full reporting of outcomes |
| ☒ | | Estimates of effect sizes (e.g. Cohen's *d*, Pearson's *r*), indicating how they were calculated |

*Our web collection on statistics for biologists contains articles on many of the points above.*

## Software and code

Policy information about availability of computer code

| | |
|---|---|
| Data collection | An ensemble of Y2R models with the disordered NT in contact with the NPY peptide was generated by AlphaFold2 version 2.1 and RosettaCM. MD Simulations were carried with GROMACS 2024.2, using the Charmm36m force field. |
| Data analysis | MS data was processed with DataAnalysis (v5.3; Bruker Daltonics, Bremen, Germany), MeroX v. 2.0.1., proXL, Skyline. MS/MS peak lists were analyzed using MS-GF+ (v 2023.01.12) and MyriMatch (v 2.2.140) using SearchGUI (v 4.2.14). NMR data were processed with Topspin 4.4.1. The software PYMOL (version 2.5.2, Schrödinger LLC), VMD1.9, NLG, MDsrv, and mdciao were used to visualize and analyze MD simulations. Cell Profiler v4.2.8 was used to quantify microscopy images. GraphPadPrism 9 was used to visualize signaling data and perform statistical analysis. |

For manuscripts utilizing custom algorithms or software that are central to the research but not yet described in published literature, software must be made available to editors and reviewers. We strongly encourage code deposition in a community repository (e.g. GitHub). See the Nature Portfolio guidelines for submitting code & software for further information.

## Data

Policy information about availability of data

All manuscripts must include a data availability statement. This statement should provide the following information, where applicable:

- Accession codes, unique identifiers, or web links for publicly available datasets
- A description of any restrictions on data availability
- For clinical datasets or third party data, please ensure that the statement adheres to our policy

XL-MS data have been submitted to ProteomeXchange with identifier PXD051865. MD-trajectories are accessible under https://proteinformatics.uni-leipzig.de/mdsrv.html?load=file://_nt/y2r_nt.js. Source data on receptor mutants and their signaling are provided with this paper. All data are available from the corresponding authors upon request.

## Research involving human participants, their data, or biological material

Policy information about studies with human participants or human data. See also policy information about sex, gender (identity/presentation), and sexual orientation and race, ethnicity and racism.

| | |
|---|---|
| Reporting on sex and gender | N/A |
| Reporting on race, ethnicity, or other socially relevant groupings | N/A |
| Population characteristics | N/A |
| Recruitment | N/A |
| Ethics oversight | N/A |

Note that full information on the approval of the study protocol must also be provided in the manuscript.

# Field-specific reporting

Please select the one below that is the best fit for your research. If you are not sure, read the appropriate sections before making your selection.

☒ Life sciences ☐ Behavioural & social sciences ☐ Ecological, evolutionary & environmental sciences

For a reference copy of the document with all sections, see nature.com/documents/nr-reporting-summary-flat.pdf

# Life sciences study design

All studies must disclose on these points even when the disclosure is negative.

| | |
|---|---|
| Sample size | Sample size were not predetermined by statistical methods. For all experiments, 2-5 independent experiments were performed in technical duplicate/ triplicate to ensure that data are reproducible. All experiments were conducted with pools of cells (>10.000 cells per sample) or purified protein in bulk concentration and the experimental outcomes are expected to follow a normal distribution. Therefore, the mean of 3-5 independent experiments is expected to be sufficiently close to the mean of the population and will follow normal distributions with reasonable accuracy. |
| Data exclusions | No data were excluded from the analyses. |
| Replication | For all experiments, 2-5 independent experiments were performed in technical duplicate/ triplicate to ensure that data are reproducible. All attempts of replication were successful. |
| Randomization | Randomization was not applicable to this study. No animals or human research participants are involved in this study. Structural (XL-MS and NMR) experiments as well as MD simulations were conducted with only the WT. In signaling experiments, all receptor variants were transiently transfected in separate wells and tested alongside the wild-type sample. |
| Blinding | Experimentators were not blinded to the samples analyzed. All data were aquired and analyzed with defined and consistent protocols to ensure objectivity. |

# Reporting for specific materials, systems and methods

We require information from authors about some types of materials, experimental systems and methods used in many studies. Here, indicate whether each material, system or method listed is relevant to your study. If you are not sure if a list item applies to your research, read the appropriate section before selecting a response.

## Materials & experimental systems

| n/a | Involved in the study |
| --- | --- |
| ☒ | ☐ Antibodies |
| ☐ | ☒ Eukaryotic cell lines |
| ☒ | ☐ Palaeontology and archaeology |
| ☒ | ☐ Animals and other organisms |
| ☒ | ☐ Clinical data |
| ☒ | ☐ Dual use research of concern |
| ☒ | ☐ Plants |

## Methods

| n/a | Involved in the study |
| --- | --- |
| ☒ | ☐ ChIP-seq |
| ☒ | ☐ Flow cytometry |
| ☒ | ☐ MRI-based neuroimaging |

# Eukaryotic cell lines

Policy information about cell lines and Sex and Gender in Research

| | |
| --- | --- |
| Cell line source(s) | HEK293 were obtained from DSMZ, ACC305 |
| Authentication | The cells were not authenticated. |
| Mycoplasma contamination | Cells were tested negative for potential mycoplasma contamination. |
| Commonly misidentified lines (See ICLAC register) | No commonly misidentified cell lines were used. |

# Plants

| | |
| --- | --- |
| Seed stocks | N/A |
| Novel plant genotypes | N/A |
| Authentication | N/A |
